# Life and death in a dynamic environment: Invasive trout, floods, and intraspecific drivers of translocated populations

**DOI:** 10.1002/eap.2635

**Published:** 2022-06-13

**Authors:** Brian D. Healy, Phaedra Budy, Mary M. Conner, Emily C. Omana Smith

**Affiliations:** ^1^ Department of Watershed Sciences and the Ecology Center Utah State University Logan Utah USA; ^2^ Native Fish Ecology and Conservation Program, Division of Science and Resource Management Grand Canyon National Park, National Park Service Flagstaff Arizona USA; ^3^ United States Geological Survey, Utah Cooperative Fish and Wildlife Research Unit, Department of Watershed Sciences Utah State University Logan Utah USA; ^4^ Department of Wildland Resources and the Ecology Center Utah State University Logan Utah USA

**Keywords:** density dependence, flooding, flow ecology, invasive species, mark–recapture, population regulation, rainbow trout, reintroduction, somatic growth, temporal symmetry model

## Abstract

Understanding the relative strengths of intrinsic and extrinsic factors regulating populations is a long‐standing focus of ecology and critical to advancing conservation programs for imperiled species. Conservation could benefit from an increased understanding of factors influencing vital rates (somatic growth, recruitment, survival) in small, translocated populations, which is lacking owing to difficulties in long‐term monitoring of rare species. Translocations, here defined as the transfer of wild‐captured individuals from source populations to new habitats, are widely used for species conservation, but outcomes are often minimally monitored, and translocations that are monitored often fail. To improve our understanding of how translocated populations respond to environmental variation, we developed and tested hypotheses related to intrinsic (density dependent) and extrinsic (introduced rainbow trout *Oncorhynchus mykiss*, stream flow and temperature regime) causes of vital rate variation in endangered humpback chub (*Gila cypha*) populations translocated to Colorado River tributaries in the Grand Canyon (GC), USA. Using biannual recapture data from translocated populations over 10 years, we tested hypotheses related to seasonal somatic growth, and recruitment and population growth rates with linear mixed‐effects models and temporal symmetry mark–recapture models. We combined data from recaptures and resights of dispersed fish (both physical captures and continuously recorded antenna detections) from throughout GC to test survival hypotheses, while accounting for site fidelity, using joint live‐recapture/live‐resight models. While recruitment only occurred in one site, which also drove population growth (relative to survival), evidence supported hypotheses related to density dependence in growth, survival, and recruitment, and somatic growth and recruitment were further limited by introduced trout. Mixed‐effects models explained between 67% and 86% of the variation in somatic growth, which showed increased growth rates with greater flood‐pulse frequency during monsoon season. Monthly survival was 0.56–0.99 and 0.80–0.99 in the two populations, with lower survival during periods of higher intraspecific abundance and low flood frequency. Our results suggest translocations can contribute toward the recovery of large‐river fishes, but continued suppression of invasive fishes to enhance recruitment may be required to ensure population resilience. Furthermore, we demonstrate the importance of flooding to population demographics in food‐depauperate, dynamic, invaded systems.

## INTRODUCTION

The relative strengths of intrinsic and extrinsic factors regulating populations are a long‐standing focus of ecological study and an important subject of debate for both ecologists and resource managers (Lobón‐Cerviá, 2014; Rose et al., 2001; Turchin, 1999). Understanding population regulation is critical for both biodiversity conservation (Strayer & Dudgeon, 2010) and sustainable management of harvested species (Hilborn et al., 1995). Defining functional relationships between demographic vital rates (i.e., survival, recruitment) and variation in extrinsic (e.g., density‐independent predation, disturbances, or harvest rates) and intrinsic (i.e., density‐dependent) factors is essential for predicting fluctuations in abundance and understanding factors limiting populations (Frederiksen et al., 2014; Morris & Doak, 2002; Nichols & Armstrong, 2012). A vast body of literature exists describing drivers of population dynamics of many economically important game or commercially harvested species for the purpose of sustainable yield calculation (e.g., Hilborn & Walters, 1992). In comparison, knowledge of causes of variation in vital rates in imperiled species’ populations, which is critical for planning and executing conservation actions, is generally lacking (Sibly & Hone, 2002). This understanding may be limited by misallocated monitoring (i.e., lack of focused monitoring directed toward understanding critical uncertainties that if known, would influence management decisions; Runge et al., 2011), the inherent rarity or behavioral characteristics of imperiled species (Folt et al., 2020), and monitoring programs consisting solely of count data or lacking long‐term data sets (Wheeler et al., 2018; reviewed in Margalida et al., 2020).

As biodiversity loss may continue to accelerate with global change, expanded and effective conservation programs, informed by knowledge of population regulation, are critical (Hoffmann et al., 2010; Reid et al., 2019; Strayer & Dudgeon, 2010). This need is especially acute for obligate freshwater species that have suffered greater declines than terrestrial or marine species (Reid et al., 2019, Strayer & Dudgeon, 2010); many freshwater species may already occur at densities below thresholds of population viability (Strayer & Dudgeon, 2010). Novel or intensifying threats to riverine biota include, but are not limited to, climate change, species invasions, and expanding water and hydropower development to meet expanding human needs (Albert et al., 2020; Reid et al., 2019; Strayer & Dudgeon, 2010). Among fishes, those inhabiting extensively fragmented arid‐ and semiarid‐land river systems are among the most imperiled (Fagan et al., 2002). Expanding needs for human water development and threats imposed by invasive species introductions into environments with severely altered flow regimes require intensive conservation actions for arid‐land species (Bond et al., 2015; Propst et al., 2008).

Reintroductions or translocations (from this point forwards translocations), defined here as the movement of individuals from one source population to another area of a species’ former range, could provide a means to recover imperiled species (reviewed in Armstrong & Reynolds, 2012), including those inhabiting dry regions (Cahn et al., 2011; Lintermans, 2013; Spurgeon, Paukert, Healy, Trammell, et al., 2015). Relative to terrestrial wildlife and birds, fewer translocations of fishes are reported in the literature (Brichieri‐Colombi & Moehrenschlager, 2016), in spite of 80% of endangered fish recovery programs in the USA including translocation as a recovery action (George et al., 2009; Williams et al., 1988). Nonetheless, translocations remain controversial given the potential to impact source populations (e.g., Lamothe et al., 2021; Pine et al., 2013), introduce disease, or cause other harmful negative impacts to the receiving ecosystems (George et al., 2009; Olden et al., 2011; Pérez et al., 2012).

Many translocations also fail, especially those involving endangered species (Griffith et al., 1989; reviewed in Cayuela et al., 2019). All too often, translocations are inadequately planned or monitored (Strayer & Dudgeon, 2010), or measurable objectives are not established to quantify and report outcomes (Galloway et al., 2016; George et al., 2009; reviewed in Sheller et al., 2006). When clear outcomes were reported, failures of translocations to establish self‐sustaining populations were related to insufficient or unsuitable habitat (Griffith et al., 1989; Harig et al., 2000), limited duration of a program, the number (i.e., propagule pressure) and genetic origin of individuals translocated, the season of release (Cochran‐Biederman et al., 2015; Sheller et al., 2006), predation by introduced fishes (Al‐Chokhachy et al., 2009), and failure to address the initial causes of decline (e.g., continued presence of nonnative species; Cochran‐Biederman et al., 2015). Assessment of demographic rates in translocated fish populations are also rare (Armstrong & Reynolds, 2012; Vincenzi, Crivelli, et al., 2012). Given the prevalence of translocations in recovery plans, a clear need exists to evaluate translocation efficacy in recovering endangered or threatened species (George et al., 2009; Minckley, 1995; Olden et al., 2011; Sheller et al., 2006), including the likelihood of persistence of translocated populations under varying environmental conditions in receiving habitats (Vincenzi, Crivelli, et al., 2012).

The context under which compensatory mechanisms confer population resilience in small translocated populations, including the drivers of variation in individual‐ or population‐level growth within and among populations, are important uncertainties to be addressed (Sibly & Hone, 2002; Vincenzi et al., 2016; Winemiller, 2005). How populations compensate for high mortality related to disturbance or losses due to invasive species predation, for example, will depend on how populations are regulated at low densities (Vincenzi, Crivelli, et al., 2012). Detection of density dependence in vital rates can also provide insights into the carrying capacity of habitats where translocations occur. Once factors regulating populations are understood, managers can prioritize actions for endangered species recovery in the context of environmental variation, and predict how small populations may respond (Conner et al., 2018; Vincenzi, Crivelli, et al., 2012). For instance, invasive species can limit populations of imperiled species through predation or competition; however, environmental conditions, including those related to changing climate, may mediate these impacts (reviewed in Rahel et al., 2008), or influence the population‐level response of native species to invasive species suppression (Healy, Schelly, et al., 2020). Attempts to repatriate species may also be thwarted by severe floods or wildfire (Hickerson & Walters, 2019; Vincenzi, Crivelli, et al., 2012). The frequency and impact of such catastrophic events must therefore be considered to understand long‐term population viability when planning conservation actions (Conner et al., 2018; Reed et al., 2003).

Monitoring that assesses vital rate relationships with environmental variables (e.g., stream flow metrics, indices of invasive species abundance) or intraspecific density are advantageous and underused for identifying the underlying mechanisms regulating demographic variation (Wheeler et al., 2018). Mark–recapture techniques allow for estimates of abundance, survival, recruitment, and temporary emigration; different configurations of these translate to a given state (i.e., abundance at time *t*) and defining the relative strength of each process in driving population growth rates can help to focus conservation (Armstrong & Reynolds, 2012; Budy et al., 2017; Wheeler et al., 2018). For example, restoring habitats in migratory routes and protecting large adults from harvest were recommended for endangered Gulf of Mexico sturgeon (*Acipenser oxyrinchus desotoi*; Pine et al., 2001) and bull trout (*Salvelinus confluentus*; Budy et al., 2017) conservation, and placement of supplementary feeding sites to reduce negative density‐dependent effects on adult survival was suggested to expand bearded vulture (*Gypaetus barbatus*; Margalida et al., 2020) populations; all species with population growth driven by adult survival.

Here, through the use of a multimark–recapture model approach, we examined demographic variation in translocated populations of a long‐lived federally endangered large‐river cyprinid, humpback chub (*Gila cypha*), inhabiting the semiarid Colorado River basin in the southwestern USA. Many native fishes of the region are imperiled due to the prevalence of dams and water diversions (Sabo et al., 2010) that fragment habitats and block migration routes (Fagan et al., 2002). Dramatically altered flow, sediment, and temperature regimes (Schmidt, 2010) in the Colorado River also limit native fish reproduction and facilitate the replacement of native fauna by introduced invasive fishes (Holden & Stalnaker, 1975; Olden et al., 2006). The largest remaining humpback chub population exists downstream of the Glen Canyon Dam within the Grand Canyon, Arizona, USA (USFWS, 2018). The 1963 construction and operation of the Glen Canyon Dam altered or eliminated humpback chub spawning habitats within Grand Canyon National Park (GCNP; Schmidt et al., 1998, Clarkson & Childs, 2000), where humpback chub face predation and competition with introduced fishes (Marsh & Douglas, 1997; Yard et al., 2011). Until recently (Healy, Omana Smith, et al., 2020; van Haverbeke et al., 2017), the Grand Canyon population was sustained almost solely by reproduction in a seasonally warm tributary, the Little Colorado River (LCR; Valdez & Masslich, 1999; reviewed in Pine et al., 2013). Managers initiated translocations to attempt to establish new populations in tributaries with more benign conditions (i.e., fewer predators, suitable thermal regimes) than in the Colorado River, in order to increase population redundancy (Healy, Omana Smith, et al., 2020; Spurgeon, Paukert, Healy, Trammell, et al., 2015) and reverse decadal‐scale declines in abundance (Coggins et al., 2006). Knowledge of drivers of demographic rates in these populations could assist managers in planning translocations and mitigating additional stressors to endangered humpback chub and other imperiled fishes.

We assessed hypothesized mechanistic relationships between temporally varying environmental factors and humpback chub somatic growth, survival, recruitment, and emigration rates. Over a 10‐year period, we studied responses in two populations of humpback chub translocated from the LCR to two small Colorado River tributaries. Specifically, our objectives were to (a) evaluate hypothesized relationships between juvenile somatic growth, recruitment, survival, and fidelity rates with invasive rainbow trout (*Oncorhynchus mykiss*) abundance and seasonally varying thermal and flow regimes; (b) assess the degree of density dependence in life‐stage specific vital rates; and (c) identify relative strengths of recruitment and survival in driving population growth rates among translocated fish and those produced in situ. We assessed evidence for the following hypothesized relationships between humpback chub vital rates and environmental drivers (additional humpback chub species information and study hypotheses are included in Appendix S1):

1. Individual growth, recruitment, and survival rates will vary with flood frequency, magnitude, timing, and duration. Growth of subadults would be constrained in winter (Dzul et al., 2016), but enhanced during summer months in years with higher frequency of floods (Behn & Baxter, 2019). We predicted young‐of‐year (YOY) recruitment (survival from birth to age‐1) would be limited during years with higher monsoon flood frequency or intensity, as in the LCR (Yackulic et al., 2014). Once recruited into the subadult or adult population, we would expect minimal effects of flooding on survival, with the exception of ash‐laden floods that may limit survival of southwestern United States fishes (Gido et al., 2019).
2. We expect density‐dependent growth and recruitment, but relationships between density and vital rates may be less important in sites with high food resources and with high emigration rates, compared with other drivers. The effects of negative density dependence are assumed to weaken with size and age in the tributary humpback chub source population (Pine et al., 2013), but previous work found only weak support for density‐dependent growth and survival in the Colorado River (Yackulic et al., 2018).
3. Rainbow trout will limit growth, survival (Yackulic et al., 2018), and ultimately recruitment of humpback chub in translocation sites, given high trophic niche overlap between the two species (Spurgeon, Paukert, Healy, Kelley, et al., 2015), and evidence of direct predation by rainbow trout upon YOY or subadult humpback chub (Yard et al., 2011).

## METHODS

### Study area

**FIGURE 1:**
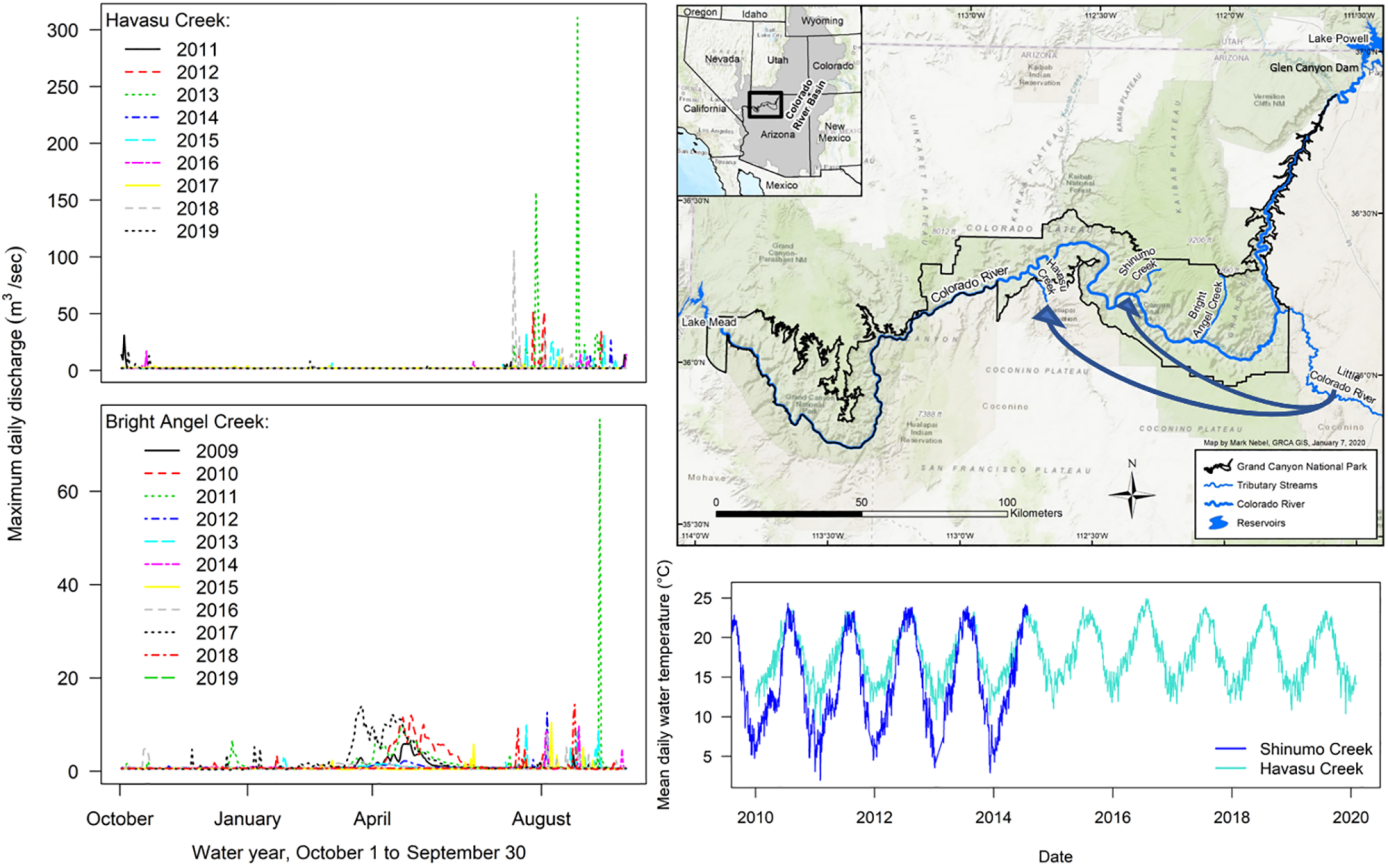
Study area, with arrows depicting translocations of humpback chub from the Little Colorado River to Shinumo and Havasu Creeks, within Grand Canyon National Park, Arizona, USA. Flow regimes (maximum daily discharge, m^3^/sec, by water year) in Havasu and Bright Angel creeks, and temperature regimes (mean daily temperature °C) in Shinumo and Havasu Creeks occurring during the duration of the study, as also displayed. Discharge data from Bright Angel Creek, an adjacent watershed to the east of Shinumo Creek, were used to calculate flood‐pulse frequency to represent conditions in Shinumo Creek.

Translocation sites were chosen in GCNP, on the semiarid Colorado Plateau, which is bisected by 446 km of the Colorado River between Glen Canyon Dam and Lake Mead reservoir (Figure 1). Havasu and Shinumo Creeks, joining the Colorado River from the South Rim and North Rim of the Grand Canyon, respectively, were prioritized for translocations following an assessment of thermal characteristics, physical habitat, and biological conditions in several tributaries (Healy, Omana Smith, et al., 2020; Spurgeon, Paukert, Healy, Trammell, et al., 2015; Valdez et al., 2000). Physical and chemical characteristics of Havasu Creek and the LCR are thought to be most similar among GCNP tributaries, as calcium carbonate precipitates form large travertine dams and step‐pools; however this unique water chemistry may also limit macroinvertebrate production (Oberlin et al., 1999), which was an order of magnitude lower in Havasu Creek relative to Shinumo Creek (Appendix S2: Figure S1).

The flow regimes in translocation sites differ; while the baseflow of both streams is driven by perennial groundwater discharge, during years with substantial snowpack at higher elevations on the North Rim, Shinumo Creek experiences spring snowmelt runoff, with intense, short‐duration (<1 day) monsoon storm‐driven flooding in summer (~July–September; Tobin et al., 2018). Havasu Creek hydrology differs, as no prolonged spring snowmelt discharge occurs. Flooding in Havasu Creek is generally associated with monsoon‐season storms, which can be intense (>280 m^3^ s^−1^) but also of short duration (Melis et al., 1996). Baseflow discharge in the fall and winter in Shinumo Creek is ~0.26 m^3^ s^−1^ (Spurgeon, Paukert, Healy, Trammell, et al., 2015), while Havasu Creek baseflow is 1.8 m^3^ s^−1^ (Figure 1; U.S. Geological Survey (USGS), gaging station 9404115; USGS, 2020b). On 28 July 2014, an intense rainstorm on a freshly burned area comprising ~10% of the Shinumo Creek watershed triggered a massive flood that carried heavy loads of ash, destroying monitoring equipment, and extirpating translocated humpback chub. Debris flows triggered by intense and localized rainfall that reorganize stream channels are also common (occurring in 18% of tributaries in 20 years) in Grand Canyon tributaries (Griffiths et al., 2004). While we lacked a long‐term hydrologic record for Shinumo Creek, we assumed a flood of the magnitude observed in 2014 was a rare event, because it destroyed historical dwellings in existence for >100 years (B. Healy, E. Omana Smith, NPS, personal observation).

Annual and seasonal variation in water temperatures (from this point forwards “temperature”) in Shinumo Creek exceeds that of Havasu Creek (Voichick & Wright, 2007), and while maximum temperatures are similar, Shinumo Creek has lower winter temperatures (Figure 1). Havasu Creek water temperatures were suitable for humpback chub growth throughout the year (>12°C, Hamman, 1982), with some exceptions, while Shinumo Creek was expected to provide seasonally suitable temperatures (Figure 1).

The fish assemblage in Havasu Creek consists primarily of native species with small numbers (averaging <2% of fish) of rainbow trout captured (Healy, Omana Smith, et al., 2020). Rainbow trout, an invasive salmonid introduced into GCNP in the 1920s (reviewed in Runge et al., 2018), was abundant in Shinumo Creek (Spurgeon, Paukert, Healy, Kelley, et al., 2015). Speckled dace (*Rhinichthys osculus*) were the most prevalent of native fishes in both streams during our study, followed by bluehead sucker (*Catostomus discobolus*), prior to extirpation from Shinumo Creek in 2014 (Healy, Omana Smith, et al., 2020; Spurgeon, Paukert, Healy, Trammell, et al., 2015). A ~3 m waterfall near the mouth of Shinumo Creek, and steep cascades near the mouth of Havasu Creek, prevents immigration of fishes from the Colorado River, with the exception of a small number of humpback chub that presumably moved into Havasu Creek during high 2011 Colorado River discharge for reservoir storage equalization (discussed in Healy, Omana Smith, et al., 2020). Historical, pre‐dam fish survey data for tributaries prior to trout introductions in GCNP are limited to anecdotal reports that contain little species‐specific information.

### Translocation process

The process of collecting, rearing, and translocating humpback chub is described in detail in Spurgeon, Paukert, Healy, Trammell, et al. (2015), and Healy, Omana Smith, et al. (2020). In summary, we collected wild YOY or juvenile humpback chub from the LCR in summer or fall months, transferred the fish to a federal or state hatchery for parasite and disease treatment. Once large enough (>80–100 mm total length [length]; please refer to Ward et al., 2015), humpback chub were tagged with a 12‐mm passive‐integrated transponder (PIT) tag. We released a total of 1002 subadult humpback chub in groups of 200–302 individuals in Shinumo Creek annually in June, between 2009 and 2013, with the exception of 2012 (Appendix S2: Table S1). In Havasu Creek, we released a total of 1955 humpback chub in groups of 243–305 in May, June, or July between 2011 and 2016. We completed translocations in both May (300 fish) and July (209 fish) of 2014 to Havasu Creek – fish destined to be released in June to Shinumo Creek were diverted to Havasu Creek in July to avoid exposing fish to potential impacts of an active fire in the Shinumo Creek watershed.

### Field methods: Translocation monitoring

Monitoring of translocated fish was conducted within translocation sites by crews led by the authors, or throughout the Colorado River ecosystem (CRE; i.e., Colorado River and its tributaries from Glen Canyon Dam to Lake Mead) during interagency monitoring associated with the Glen Canyon Dam Adaptive Management Program (GCDAMP). Sampling protocols for monitoring translocated populations are described in Healy, Omana Smith, et al. (2020) and Spurgeon, Paukert, Healy, Trammell, et al. (2015). In general, we monitored translocated populations during biannual hoop‐netting events conducted in spring or summer (pre‐monsoon season) and fall (post‐monsoon) of each year, with two netting passes throughout reaches accessible to translocated fish at least once per year. We were forced to cancel one planned monitoring event in Havasu Creek during the suspension of United States Government operations during October 2013, and sampling was disrupted by a late monsoon season Havasu Creek flood in October 2018. However, we consistently monitored between June 2009 and September 2014 in Shinumo Creek, and from June 2011 through October 2019 in Havasu Creek. Humpback chub dispersing from translocation sites were recaptured throughout the CRE during standardized river‐wide electrofishing or hoop‐netting administered through the GCDAMP (described in van Haverbeke et al., 2017; Rogowski et al., 2018), or in the LCR (please refer to van Haverbeke et al., 2013 for details). Additional hoop‐netting focused on the Havasu and Shinumo Creek inflow reaches of the Colorado River was also conducted consistently under the GCDAMP beginning in 2010 (Persons et al., 2017) or by our sampling crews after 2013 (~60 net sets per trip; Shinumo only). Handling and processing of native and invasive fish followed standardized protocols established for GCNP (Persons et al., 2013). We generally avoided tagging humpback chub <100 mm and those 100–150 mm in length engorged with bait to minimize perforation of the gut and potential mortality (distribution of size of fish at tagging; Appendix S2: Figure S2).

We used continuously collected PIT‐tag detection data from fixed passive interrogation antennae (PIAs) established prior to translocations in Shinumo Creek (June 2009–July 2014), in Bright Angel Creek (May 2018–present), and in the LCR to augment capture histories for survival models (described below). PIAs in Shinumo and Bright Angel creeks spanned the width of their respective stream channels, and were installed as close to the mouth as possible (~200 m), but differed in the number of arrays; Shinumo Creek consisted of two antenna arrays installed 200 m upstream of the waterfall, while three were installed in Bright Angel Creek for additional redundancy to improve detection rates. PIA operations were uninterrupted with some exceptions; due to power supply issues and flood damage, the LCR PIA has operated intermittently since 2009 (Pearson et al., 2015) with more continuous operation between August 2017 and August 2019, and the Shinumo Creek PIA power failed briefly during winter of 2010. The Shinumo PIA was destroyed during the flood of July 2014. We determined that powering an antenna array at Havasu Creek was infeasible due to site characteristics, and relied solely on recaptures in the Creek and Colorado River to populate encounter histories for Havasu Creek fish. Beginning in 2014, the GCDAMP agencies began to deploy baited, portable PIAs during river‐wide monitoring excursions that also provided detections of translocated fish. The spatial and temporal distribution of sampling effort generating data for our study is depicted in Appendix S2: Figure S3.

#### Environmental and biological predictors

We calculated physical and biological variables to test hypothesized relationships with translocated humpback chub vital rates (Table 1). Stream flow metrics represented flood duration, magnitude, timing, and frequency, which are thought to drive the population dynamics of many stream fishes (Gido et al., 2013; Poff & Ward, 1989; Richter et al., 1996). Given differences in data availability and stream discharge characteristics, stream flow metric calculation varied between streams. Large flood events can rise and fall quickly within a day, and may not be detectable when 15‐min flow records are averaged over a day. Therefore, we used instantaneous maximum daily stream flow (m^3^/s) from a USGS gaging station located near the mouth of Havasu Creek (USGS gaging station 9404115; USGS, 2020b), and lacking a continuous hydrograph for Shinumo Creek, from a nearby gage on Bright Angel Creek (USGS data, gaging station 9403000; USGS, 2020a) subjected to similar regional‐scale seasonal and annual climatic patterns (Tillman et al., 2020). We assumed that Bright Angel Creek baseflow and spring discharge were representative of seasonal hydrologic variation in Shinumo Creek, because the hydrology of both watersheds is driven by discharge from the same aquifer (e.g., synchronous spring snowmelt timing and magnitude; Tobin et al., 2018). Available daily discharge in both streams between January through June (2010–2016) was correlated (*p <* 0.001, *R* ^2^ = 0.93; Appendix S2: Figure S4); however, spring discharge magnitude can be an order of magnitude higher in Bright Angel Creek. We assumed Shinumo Creek monsoon season flood frequency, but not magnitude and duration, would be represented by Bright Angel Creek discharge data. Therefore, for Shinumo Creek, we defined flood‐pulse frequency as the number of days the maximum daily flow exceeded two standard deviations greater than baseflow (>2.8 m^3^/s), calculated from the stream flow record corresponding to our study period (Resh et al., 1988; Richter et al., 1996) in Bright Angel Creek.

**TABLE 1:** List of biological, hydrological, and other variables and their abbreviations used in figures, along with each variables’ hypothesized relationship with humpback chub demographic rates

Variables	Abbreviation	Hypothesized effect	Analyses
Biological variables			
Humpback chub catch‐index of abundance	HBC.catch	Density dependence	Growth, survival–fidelity, recruitment (Havasu only)
Number of humpback chub translocated	No.Transl	Density dependence	Growth, survival–fidelity
Total length of individual (mm)	Total length	Declining growth rate with size	Growth
Rainbow trout catch‐index of abundance	RBT.catch	Predation/competition	Growth, survival–fidelity, recruitment (Havasu only)
Speckled dace catch‐index of abundance	SPD.catch	Food base indicator	Survival–fidelity
Hydrology variables			
Flood‐pulse frequency (number of days discharge > 2.8 m^3^/s)	Floodpulse	Flood frequency/duration	Growth, survival–fidelity, recruitment (Havasu only)
Number of days of flooding > 28 m^3^/s	days.ov.1000	Flood magnitude/duration, large disturbance/displacement	Survival–fidelity
Maximum flood size during interval	max.Flood	Flood magnitude/timing, disturbance	Survival–fidelity
Number of days following a translocation before flood > 28 m^3^/s occurs	No. days to 1000	Flood timing/magnitude – large disturbance/displacement	Survival–fidelity
Other variables			
Season: summer or winter	Season	Represents seasonal differences in stream productivity and energetic demands	Growth, survival–fidelity
Stream (Havasu or Shinumo creeks)	Stream	Represents differences in intrinsic conditions in translocation sites not captured by other variables	Growth
Acres of fire burned below the Canyon rim (Shinumo only, fires occurred in 2010, 2011, 2014)	Fire_brim	Ash limits survival	Survival (Shinumo only)
Temperature: cumulative degree days (base 10°C)	cDD	Temperature effect	Growth, survival–fidelity

Our approach to calculating Havasu Creek flow metrics differed from Shinumo Creek, given the rare and intense nature of floods (Melis et al., 1996), lack of spring snowmelt runoff, and availability of a complete flow record (USGS gaging station 9404115; USGS, 2020b). The number of days flooding exceeded 2.8 m^3^/s, and the number of days discharge exceeded 28 m^3^/s for each interval between sampling events captured variation in flood frequency and magnitude, in addition to the maximum (peak) flow in each season. We calculated the number of days between translocations and the occurrence of a flood >28 m^3^/s to understand how the timing of large floods following translocations would impact survival and fidelity (Table 1).

We represented seasonal (summer, winter) temperature variation in our models as cumulative degree days (CDD; 10°C base; Chezik et al., 2014) calculated from mean daily temperatures measured at the Havasu Creek gaging station, and from a temperature logger placed near the mouth of Shinumo Creek recording at hourly intervals through the duration of our study. Summer and winter CDD were calculated between the first days of each spring and fall sampling event (e.g., between spring 2012 and fall 2012, and between fall 2012 and spring 2013, etc.).

We included biological variables in our models representing indices of abundance of humpback chub, speckled dace, and rainbow trout. We used the total catch of speckled dace, rainbow trout, and humpback chub during each sampling event at Shinumo Creek, and the total catch of humpback chub (including untagged fish) on the first sampling pass from Havasu Creek to account for differences in effort between spring and fall sampling (single vs. two‐pass sampling). We included the number of humpback chub translocated (at time *t* − 1) as another measure to test for hypothesized density‐dependent effects on vital rates.

### Data analysis

#### Modeling drivers of individual growth

We used linear mixed‐effects models (Dzul et al., 2017; Gelman & Hill, 2009; Weisberg et al., 2010) to evaluate combinations of predictors of individual somatic growth rates for summer and winter seasons of the first year following translocation of each cohort of humpback chub. We calculated individual growth rates for the 2013 Shinumo Creek cohort using the formula: growth _season_ = length_time−2_ – length_time−1_/Δ‐day (Healy, Omana Smith, et al., 2020; Spurgeon, Paukert, Healy, Trammell, et al., 2015), to maintain consistency with published growth rates for juvenile humpback chub translocated to Shinumo Creek from 2009 to 2011 (Spurgeon, Paukert, Healy, Trammell, et al., 2015), and Havasu Creek between 2011–2016 (Healy, Omana Smith, et al., 2020), minus the 2013 Havasu Creek cohort (no data available in fall 2013). To avoid potential autocorrelation related to repeated measures of PIT‐tagged individuals and assess the strength of temporally variable environmental or biological fixed effects in predicting growth rates, we included random intercepts representing each individual humpback chub and the year of the interval in all models (Weisberg et al., 2010). We provide additional details and equations defining growth models in Appendix S2.

#### Growth model selection

We tested for effects of between‐ and within‐stream temporal variation in temperature, flood‐pulse frequency, and density dependence on growth rates using combinations of covariates (Table 1) in models incorporating all cohorts from both streams. We included a categorical variable representing Shinumo and Havasu Creeks in these models. We also separately evaluated the relationship between rainbow trout abundance and humpback chub growth rates, along with other covariates, within Havasu and Shinumo Creeks (Appendix S2: Table S2). Prior to model fitting, we examined Pearson's (*r*) correlation coefficients between covariates and excluded covariates with correlations >0.70 to minimize inflated variance and difficulties in detecting effects (Dormann et al., 2013; Zuur et al., 2010). In cases in which correlations between variables that we deemed important for hypothesis testing exceeded this *r* threshold, we substituted another ecologically similar variable. We included a categorical factor variable representing season in lieu of temperature, and avoided including humpback chub and rainbow trout abundance in the same model. To assess the potential for intraspecific density‐dependent growth, and constraints on growth related to competition with trout, we included humpback chub and rainbow trout abundance covariates indexed at the end of each growth interval in models. Our base model, onto which we added other covariates, included fixed effects of season and individual fish length, measured prior to release, to account for declining growth rates with size (Pine et al., 2017). In addition to additive models, we included two‐way interactions between flood‐pulse frequency and season, as well as between humpback chub or rainbow trout abundance and flood‐pulse frequency and season, in other candidate growth hypothesis models.

We also calculated the variance inflation factor (VIF) for each of our top‐ranked models using the *car* package in R (Fox & Weisberg, 2014; R Core Team, 2019). We replaced interactions with additive terms for VIF testing. In cases in which collinearity was evident or VIF > 3, we closely examined the effect of removing individual variables on collinearity (i.e., sensitivity of coefficient and SE estimates; Zuur et al., 2010); further diagnostic procedures are described in Appendix S2. Predictors were *z*‐scored to aid in interpretation of partial regression coefficients (Gelman & Hill, 2009). We constructed all growth models using the *lme4* package in R (Bates et al., 2015; R Core Team, 2019), ranked models using AIC_c_ (Burnham & Anderson, 2002) calculated with the *bblme* package (Bolker and Team, 2017), and used *R* ^2^ calculated for the fixed effects in the models using the *sjplot* package (Lüdecke, 2019) for model comparison.

#### Survival and fidelity

We used a joint live‐recapture/live‐resight (JLRR) model to estimate survival (probability of survival through interval *i*) and site fidelity (*F*_*i*_, probability of remaining in tributaries) of translocated humpback chub (Barker, 1997). This model is particularly useful for determining the fate of translocated individuals because it can incorporate continuously collected data from PIAs and captures throughout the CRE during GCDAMP‐interagency monitoring, which we considered “resights,” as well as recaptures during targeted monitoring within translocations sites (e.g., Horton & Letcher, 2008, Conner et al., 2015). Additional parameters estimated by the JLRR model include recapture probability (*p*_*j*_) during translocation site monitoring events, resight probability outside of translocations sites (*R*_*i*_, i.e., probability of detection, given the individual survives through interval *i*), temporary emigration (*F′*_*j*_, the probability a fish is not available for capture during *j* sampling event, but is available at *j* + 1), the probability of resighting prior to death (*R′*_*i*_, probability of detection before an individual dies during the interval *i*), and the probability an animal is found dead during the interval (*r*_*i*_). We set *r*_*i*_ = 0, because only five individuals (<0.002% of translocated fish) were found dead during our study, and we assumed permanent emigration (*F′* = 0) due to the presence of barriers near the mouths of both tributaries (Spurgeon, Paukert, Healy, Trammell, et al., 2015; please refer to Healy, Omana Smith, et al., 2020). For the JLRR model, we included recaptures during summer and fall netting events between June 2009 and June 2014, and June 2011 and October 2019, for Shinumo and Havasu Creeks, respectively. Resights from GCDAMP monitoring trips between June 2009 and August 2019 from anywhere in the CRE, and resights from the Shinumo PIA between recapture events, were also included in encounter histories. Following the extirpation of humpback chub from Shinumo Creek in July 2014, zero recaptures occurred, but we created “dummy” post‐flood recapture events with fixed *p* = 1, assuming certainty of humpback chub extirpation. We also defined two groups (*g*) of humpback chub in Havasu Creek; translocated and non‐translocated fish (either fish produced *in situ*, or immigrated during elevated 2011 Colorado River discharge; Healy, Omana Smith, et al., 2020).

**FIGURE 2:**
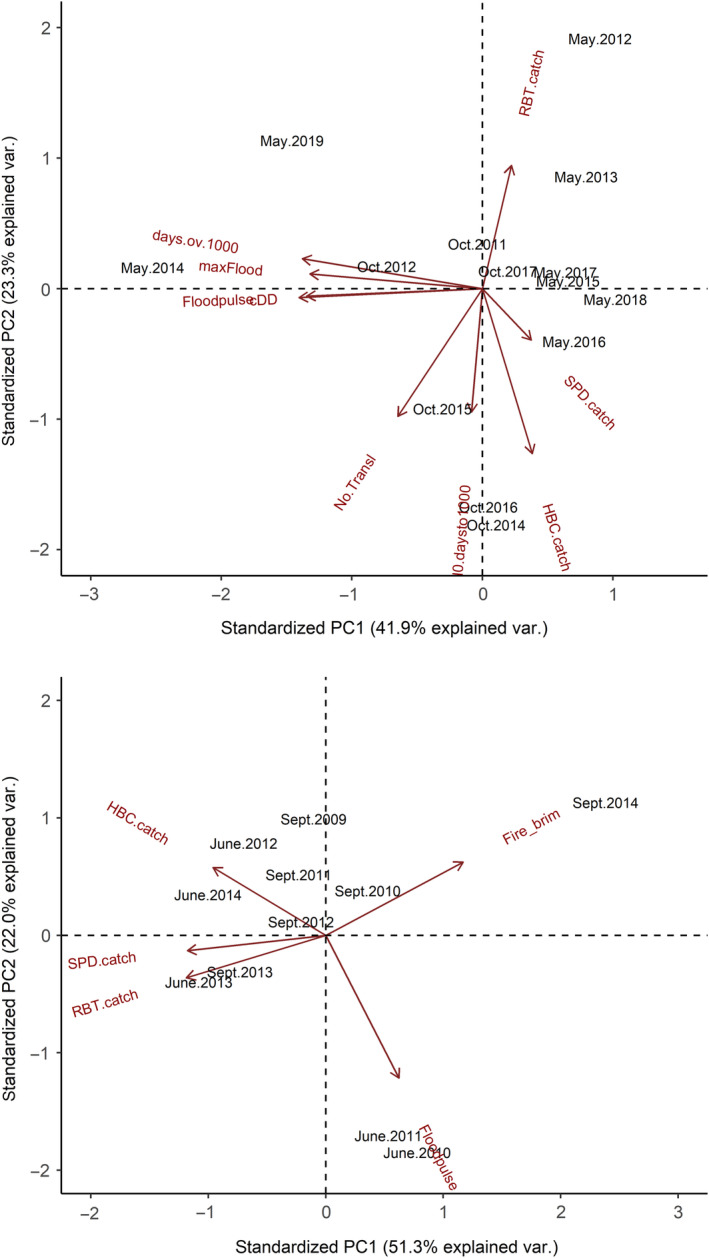
Principal components analysis scores for Havasu Creek (top) and Shinumo Creek (bottom) environmental covariates used in joint live‐resight/recapture models for survival–fidelity. Codes for each covariate are listed in Table 1.

Due to the large number of potential combinations of parameters, our JLRR model selection process proceeded in stages, which is described in detail in Supporting Information (Appendix S2). In summary, we began by finding the best‐supported structure on recapture and resight probabilities (*p*, *R*, *R′*) using combinations of time‐varying and constant parameters, and then compared combinations of models with time‐varying (*t)*, constant, and group‐specific fidelity and then survival. Finally, we combined the most‐supported model structure for *p*, *R*, *R′*, survival, and fidelity, and if top‐ranked models included *t*, we added combinations of environmental and biological covariates to survival and fidelity parameters (replacing *t* from the base model). For each translocation site, covariates consisted of two synthetic variables (PC1 and PC2) constructed using principal components analysis (PCA, Graham, 2003), with the *prcomp* function and default rotation in the *stats* package in R (R Core Team, 2019). We determined PCA to be advantageous over other multivariate methods given the underlying linear trends in our continuous variables, which we centered and standardized (i.e., PCA based on a correlation matrix) due to the differing scales of variables (Kenkel, 2006). PC1 and PC2 represented 41.9% and 23.3%, of environmental and biological variation in Havasu Creek, and 51.3% and 22.0% in Shinumo Creek (Figure 2). For Havasu Creek, PC1 represented variation in flood magnitude and frequency and temperature (−, i.e., greater flood magnitude and temperature negatively associated with PC1), and PC2 represented indices of abundance for humpback chub and the number of translocated chub (−), rainbow trout abundance (+), and the timing of large (>28 m^3^/s) floods relative to translocation timing (−, Figure 2). PC1 for Shinumo Creek represented a gradient of rainbow trout, speckled dace, and humpback chub abundance (−), and the total acres of fire below the canyon rim in the watershed (+). Shinumo Creek PC2 represented flood‐pulse frequency (−). For Shinumo Creek, we also tested whether survival differed before and after the 2014 fire and large flood event. We constructed and ranked models using Program MARK (White & Burnham, 1999) and Akaike Information Criterion adjusted for small sample sizes (AIC_c_, Burnham & Anderson, 2002).

#### Recruitment

We used a temporal symmetry model (TSM; Pradel, 1996) to assess drivers of annual recruitment rates for humpback chub in Havasu Creek. The TSM is an open‐population model that simultaneously estimates apparent survival (φ, confounded by emigration) using individual encounter histories, and estimates the relative contributions of adult survival and recruitment (*f*) toward the population growth rate (λ) that is interpretable through a “seniority probability” (γ) parameter (Budy et al., 2017; Nichols et al., 2000; Pradel, 1996). In the TSM, recruitment is defined as the number of new adults at time *t* + 1 relative to the number of adults at time *t*, and we considered newly PIT‐tagged fish as recruits. Recruits averaged the approximate length ($\bar{X}$ = 204 mm) when fish begin to mature (i.e., defined as fish in spawning condition; size at tagging; figure S2 Healy, Omana Smith, et al., 2020). For unbiased estimates of *f*, the size of the study area and sampling effort are held constant (Williams et al., 2002). We restricted our TSM analysis to data collected during spring trips when two sampling passes were consistently conducted.

We were interested in TSM estimates of λ, *f*, and γ for non‐translocated fish only (*f* of translocated fish could be regulated by additional translocations), which we separated from translocated cohorts by defining representative groups in the encounter history matrix. Assigning individuals to groups (translocated and non‐translocated) allowed us to share *p*_*j*_ from both groups if appropriate (i.e., if no group‐level differences in *p*_*j*_ were found), while generating group‐specific estimates of *f* and γ. We used the φ*fp* and the φγ*p* parameterizations of the TSM in Program MARK (White & Burnham, 1999) to construct models with all combinations of group, constant, and time‐varying φ, *p*, and *f*, to assess the relative contributions of φ and *f* to population growth. We considered estimates of γ > 0.5 to indicate greater influence of *f* on λ, while γ < 0.5 indicated that φ was more important for λ in a given year (Budy et al., 2017).

Given constraints related to annual time intervals and our inability to differentiate between seasonal variation, we limited our hypothesis testing to annual drivers of *f* during early life stages. We tested covariates including flood‐pulse frequency, and humpback chub and rainbow trout abundance indices during the natal year, as drivers of *f*, using the top‐ranked model (ranked using QAIC_c_; please refer to Appendix S2) without covariates described above. The humpback chub abundance index metric differed slightly from the metric used for survival hypothesis testing, in that we summed the number of humpback chub translocated and captured in the spring of the natal year for each cohort, which we defined as *f* year *t* – 2. The number of rainbow trout captured in spring, and flood‐pulse frequency during the summer of the natal year were also tested.

## RESULTS

All cohorts of humpback chub translocated to Shinumo Creek in 2009–2011 and 2013, and to Havasu Creek in 2011–2016, were represented in recapture data collected during monitoring events conducted in both streams between 2009–2014 and 2011–2019, respectively. We detected 51% and 38% of all fish translocated to Havasu Creek and Shinumo Creek. Through May of 2019, we also captured and tagged 232 non‐translocated humpback chub in Havasu Creek that were produced *in situ* or immigrated during 2011, but we did not capture unmarked humpback chub in Shinumo Creek upstream of Shinumo Falls.

### Individual growth modeling

**FIGURE 3:**
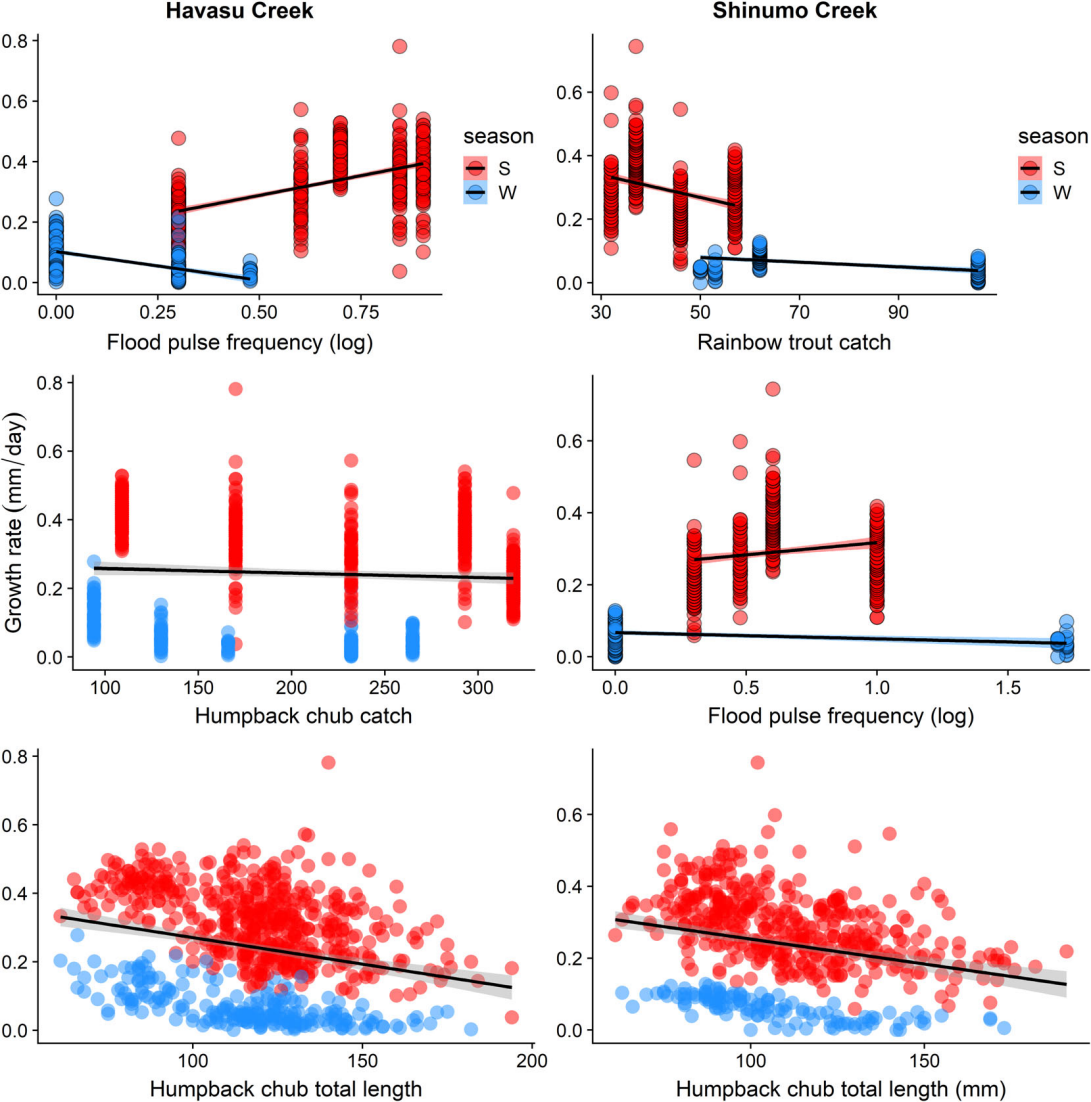
Seasonal somatic growth model results (red points = summer, blue = winter) from the top models for Havasu Creek (left column) and Shinumo Creek (right column), including relationships between daily growth rates and flood‐pulse frequency, intraspecific densities, and the interaction between season and rainbow trout abundance (upper right).

The top growth model including all cohorts had all the support (Akaike weight = 1.0, ΔAIC_c_ >10, Burnham & Anderson, 2002 **)**. The top model indicated there were lower growth rates of humpback chub in winter (range 0–0.28 mm/day) compared with summer (0.04–0.78 mm/day), lower growth rates in Shinumo Creek (0–0.74 mm/day) relative to Havasu Creek (0–0.78 mm/day), and a negative relationship with humpback chub abundance and individual length (Table 2; fixed effects *R* ^2^ = 0.86). Top models for Shinumo and Havasu Creeks suggested that humpback chub growth rates were related to humpback chub abundance and flood‐pulse frequency or rainbow trout abundance, and there were interactions between flood‐pulse frequency and season or rainbow trout abundance (Figure 3 and Table 2). The top growth model for Havasu Creek indicated growth rates were lower with higher humpback chub density, and decreased or increased in winters and summers, respectively, with higher flood‐pulse frequency (i.e., flood‐pulse frequency × season interaction; *R* ^2^ = 0.84; Table 2 and Figure 3). We observed little support for other models explaining variation in growth at Havasu (Akaike weight = 0.88, ΔAIC_c_ > 4.8); however, three models were supported explaining growth rates in Shinumo Creek (ΔAIC_c_ < 2, Akaike weights = 0.36, 0.19, and 0.16; Table 2). Rainbow trout abundance, season, flood‐pulse frequency, and a rainbow trout × season interaction explained variation in growth rates in the top‐ranked Shinumo Creek model (*R* ^2^ = 0.67, Table 2). Growth in Shinumo Creek was higher during summers with more frequent flood pulses, but growth declined with higher trout abundance during summer intervals (Figure 3). Humpback chub abundance coefficients in the 2nd and 3rd ranked Shinumo Creek growth models had weak effects (large SEs). Model fit diagnostics are included in Supporting Information (Appendix S2: Figures S5–S7).

**TABLE 2:** Humpback chub growth model results for models incorporating growth rate (dependent variables) and environmental data (predictors) from all translocated cohorts, Havasu Creek, and Shinumo Creek

Model	Intercept	Stream	Season	Total length	Humpback chub catch	Rainbow trout catch	Flood‐pulse freq.	Interaction	Akaike weights	*R* ^2^
All cohorts/streams	0.77 (0.04)	−0.5 (0.01)	−0.31 (0.001)	−0.001 (<0.001)	−0.11 (0.011)	…	…	…	1.0	0.86
Havasu Creek	0.72 (0.02)	…	−0.24 (0.01)	−0.002 (<0.001)	−0.01 (0.004)	…	0.14 (0.014)	Flood × Season −0.07 (0.01)	0.88	0.84
Shinumo Creek	0.67 (0.02)	…	−0.22 (0.01)	−0.001 (<0.001)	…	−0.11 (0.02)	0.03 (0.004)	RBT catch × Season −0.06 (0.01)	0.36	0.67
Shinumo Creek	0.65 (0.03)	…	−0.30 (0.01)	−0.001 (<0.001)	−0.007 (0.02)	…	…	HBC catch × Season −0.01 (0.01)	0.19	0.65
Shinumo Creek	0.67 (0.04)	…	−0.32 (0.02)	−0.0005 (<0.001)	0.02 (0.03)	…	−0.01 (0.008)	HBC catch × Season −0.04 (0.02)	0.16	0.64

*Note*: Partial regression coefficients (standard errors in parentheses), Akaike weights, and the coefficient of variation (*R* ^2^) are displayed for the top models (within ΔAIC_c_ < 2) for growth rates in each stream and both streams combined. HBC, humpback chub; RBT, rainbow trout.

### Survival and fidelity

**FIGURE 4:**
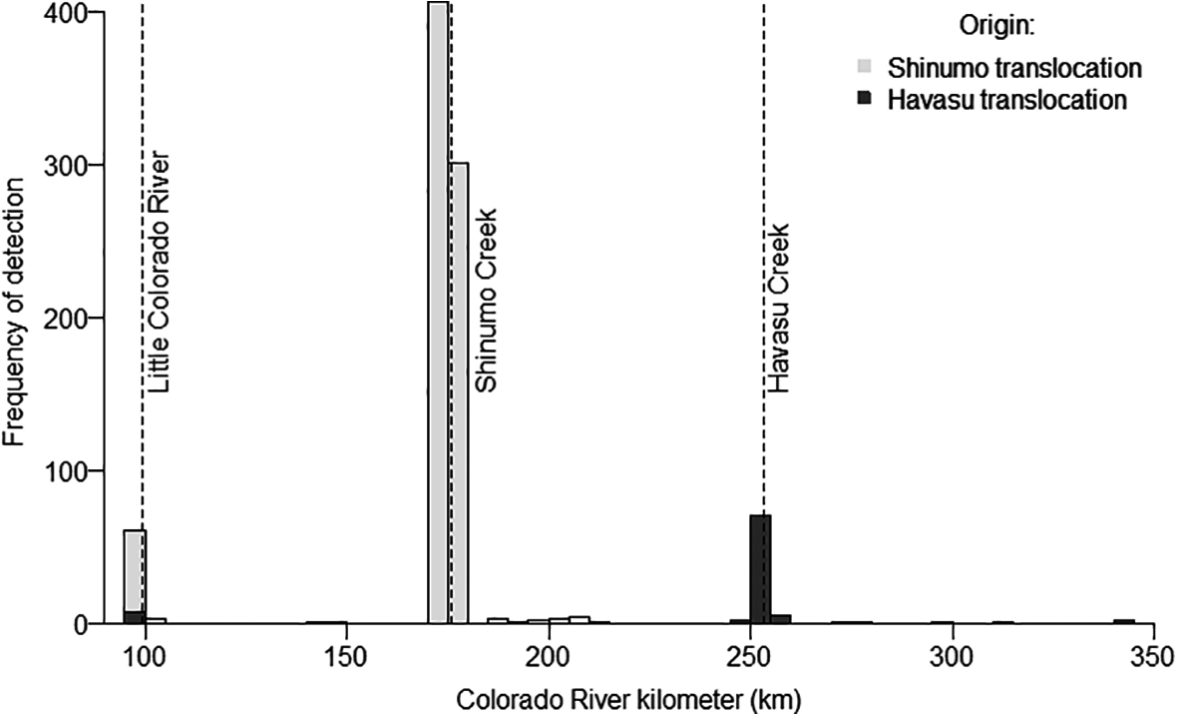
Frequency of detections of translocated fish, dispersed from Shinumo (228 of 1102 unique fish or 21%) or Havasu (73/1954 or 4%) creeks, by Colorado River kilometer (km). Fish translocated to Havasu Creek were resighted outside of Havasu Creek in the Colorado River (72 total) or the LCR (two total; one of which was also detected in the Colorado River). Upon leaving translocation sites, humpback chub dispersed upstream and downstream in the Colorado River; maximum dispersal distances from Shinumo Creek were 77 km upstream, and 34 km downstream, while fish from Havasu dispersed up to 154 km upstream through the Colorado River to the LCR and 89 km downstream. Detections include those of portable or fixed antennae or physical recaptures (i.e., netting or electrofishing) throughout the Colorado River ecosystem between Glen Canyon Dam (km −24) and Lake Mead (km 450). The river km of the confluence of tributaries where detections occurred outside of translocation sites are displayed. Dashed lines indicate the confluences of key tributaries.

In total, 767 (76%) fish translocated to Shinumo Creek were resighted at the Shinumo Creek antenna array, and 21% of 1102 fish released in Shinumo Creek were resighted in the Colorado River (228 total), LCR (12 total; nine fish were detected in both CR and LCR), or in Bright Angel Creek, where a single fish was detected on the Bright Angel Creek PIA (4 June 2019); 4% of fish translocated to Havasu Creek were detected in the Colorado River (72) or LCR (two individuals; Figure 4). In total, 11 of 232 humpback chub tagged in Havasu Creek (i.e., produced *in situ*) were resighted in the Colorado River.

**FIGURE 5:**
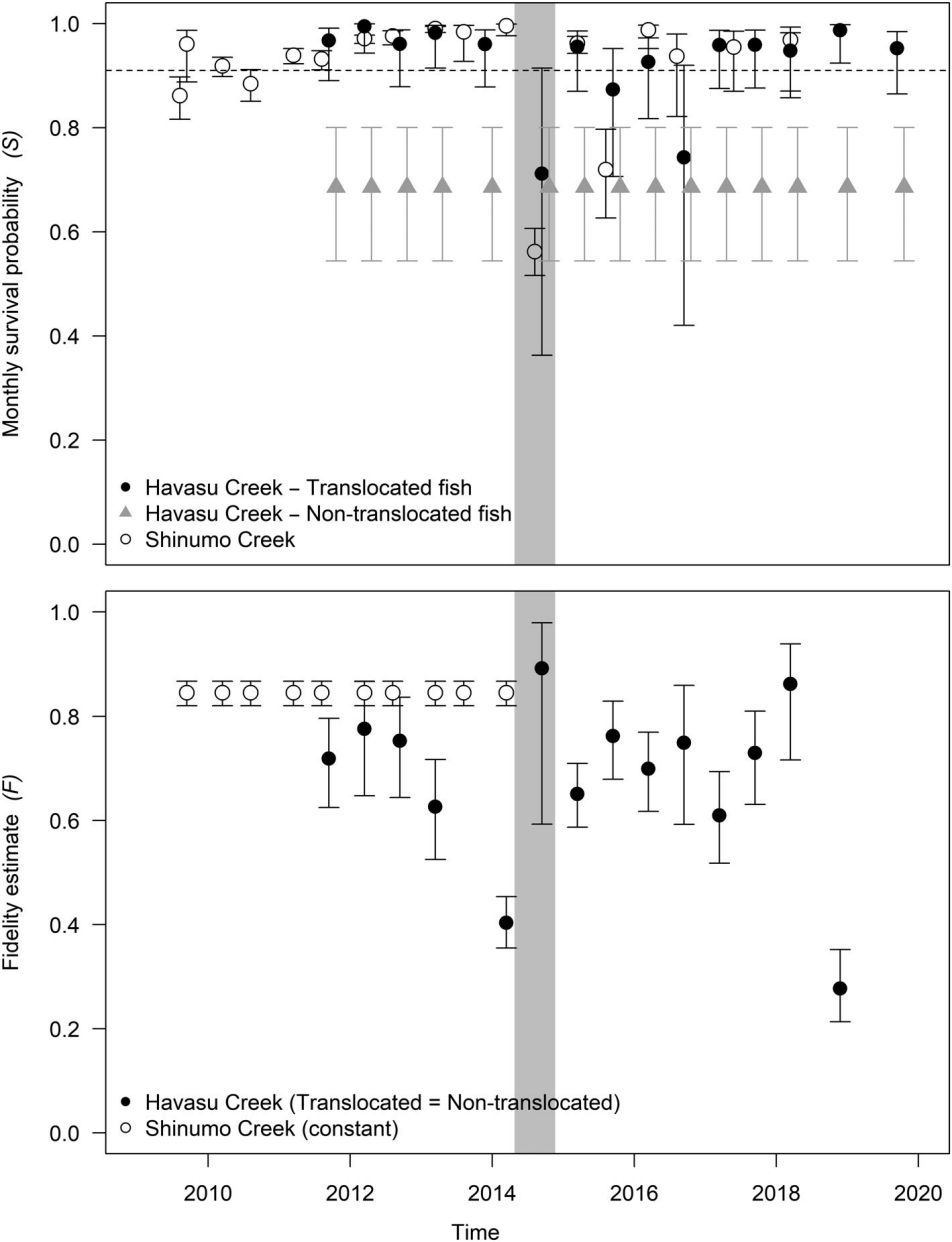
Joint live‐recapture–resight model monthly survival (upper) and fidelity (lower) estimates (with 95% confidence intervals) for humpback chub translocated to Shinumo and Havasu Creeks, and non‐translocated humpback chub initially tagged in Havasu Creek. On the survival plot, the dashed horizontal line indicates estimates of survival of small subadult humpback chub (total length 100–150 mm) in the Little Colorado River (translocation source population) 2009–2012 (Yackulic et al., 2014), and the interval corresponding to the Shinumo Creek ash‐laden flood in August 2014 is denoted by the vertical gray band. Resight and recapture probability estimates are included in Supplementary Information (Appendix S2: Figure S7).

Our JLRR results for Havasu Creek humpback chub indicated that survival differed between groups, and that survival of translocated fish was negatively density dependent (PC2) and positively associated with flooding and temperature (PC1). The top‐ranked model included time‐varying survival of translocated fish (range 0.71–0.99/month) that was a function of PC2 (0.80, SE = 0.31), constant survival of non‐translocated fish (0.69), and time‐varying fidelity (range 0.40–0.89) with no difference between groups (Table 3, Figure 5). Recapture probability (*p)* varied over time (0.47–0.89), as did resight probability (*R*, ~0–0.10), and the probability a tagged fish was resighted in the interval prior to death (*R′*) was constant in the top model (*R′* = 0.02; Appendix S2: Figure S8). There was almost equal support (Akaike weight = 0.41, model likelihood = 0.80, Table 3) for a model with the same structure on fidelity, *p*, *R*, and *R′*, as in the top‐ranked model, with survival as a function of both PC1 and PC2. The confidence interval on the PC1 coefficient (−0.67) overlapped zero (SE = 0.57, 95% confidence interval −1.80 to 0.45) but, nonetheless, models including these covariates reduced AIC_c_ by >7 when compared with the time‐varying survival model without covariates in the model. In both models, survival was lower in non‐translocated fish (survival = 0.69, 95% confidence interval 0.54–0.80). Survival was also reduced for translocated fish when humpback chub catch was greatest during summer 2014 to 2016 intervals following the largest translocation event (2014), but increased during intervals with higher flood‐frequency intensity and temperature (Figure 5). While no covariates were retained on fidelity in the top models, the lowest fidelity estimates were observed during intervals corresponding with the largest maximum flood events during the monsoon seasons of 2013 and 2018 (Figure 5, please refer to Figure 1).

**TABLE 3:** Model selection results for survival and fidelity (JLRR models) for Havasu and Shinumo Creek humpback chub, and for TSM model (apparent survival, recruitment, population growth rates and seniority) for Havasu Creek

Model	ΔAIC_c_/ΔQAIC_c_	AIC_c_ weights	Model likelihood	No. parameters	Deviance
Havasu Creek: survival and fidelity (JLRR model)					
*S*(*g*1(PCA2) *g*2(.) *p*(t) r = 0 *R*(*t*) *R*′(.) *F*(t) *F′* = 0	0	0.51	1	44	1652.97
*S*(*g*1(PCA1 + PCA2) *g*2(.) *p*(t) r = 0 *R*(*t*) *R*′(.) *F*(t) *F′* = 0	0.42	0.41	0.81	45	1651.37
*S(g*1(PCA1) *g*2(.) *p*(t) r = 0 *R*(*t*) *R*′(.) *F*(t) *F′* = 0	3.95	0.07	0.14	44	1656.92
*S*(*g*1(*t*) *g*2(.) *p*(t) r = 0 *R*(*t*) *R*′(.) *F*(t) *F′* = 0	7.33	0.01	0.03	56	1636.05
Shinumo Creek: survival and fidelity (JLRR model)					
*S*(*t*) *p*(*t*, years>2014 = 1) *r* = 0) *R*(*t*) *R′*(*t*) *F*(.)pre‐flood/*F*(.)post‐flood *F′* = 0	0	0.99	1	67	2993.17
*S*(*t*) *p*(*t*, years>2014) *r* = 0) *R*(*t*) *R′*(t*) F*(*t*)pre‐flood/*F*(.)post‐flood *F′* = 0	16.54	<0.001	<0.001	75	2948.86
*S*(pre‐flood(*t*) *S*(post‐flood(.)) *p*(*t*, years>2014) *r* = 0) *R*(*t*) *R′*(*t*) *F*(*t*) *F′* = 0	22.39	<0.001	0	75	2954.70
*S*(*t*) p(*t*) *r* = 0) *R(t) R′(t) F(t) F′* = 0	29.26	0	0	82	2947.04
Havasu Creek: recruitment (TSM model)					
φ (*t*) *p*(*t*) *f*(*g*1(*t*)*g*2 (RBT catch +Chub natal period))	0	0.36	1	25	196.59
φ (*t*) *p*(*t*) *f*(*g*1(*t*)*g*2 (RBT catch +Chub +Flooding natal period))	1.63	0.16	0.44	26	196.19
φ (*t*) *p*(*t*) *f*(*g*×*t*)	1.89	0.14	0.39	29	190.35
φ (*t*) *p*(*t*) *f*(*g*1(t)*g*2 (RBT catch natal period))	2.08	0.13	0.35	24	200.71
φ (*t*) *p*(*t*) *f*(*g*1(*t*)*g*2 (RBT catch + Flooding natal period))	2.78	0.09	0.25	25	199.37
φ (*t*) *p*(*t*) *f*(*g*1(*t*)*g*2 (Chub natal period))	3.27	0.07	0.19	24	201.89
φ (*t*) *p*(*t*) *f*(*g*1(*t*)*g*2 (Chub +Flooding natal period))	4.07	0.05	0.13	25	200.66
φ (*t*) *p*(*t*) *f*(*g*1(*t*)*g*2 (Flooding natal period))	9.45	<0.01	0.01	24	208.07

*Note*: The top‐ranked models supported by AIC_c_ for JLRR models and QAIC_c_ for TSM models (model weights ≥0.01, or top 4), are displayed. JLRR model annotation, *S*, survival; *p*, recapture probability; g, group membership (translocated or non‐translocated); *t*, time‐varying; *r*, probability of dead recovery; *R*, resight probability; *R′*, probability of detection before an individual dies during the interval; (.), constant; *F*, site fidelity; *F′*, the probability a fish is not available for capture or temporary emigration; TSM specific annotation, φ, apparent survival; *f*, recruitment rate; RBT, rainbow trout; Num. Par, number of parameters. Refer to the section *Data Analysis* for additional model details.

We conducted separate post hoc tests of individual covariates comprised of PCs 1 and 2 (Appendix S2: Table S3) in an attempt to understand the relative importance of each composite environmental effect on survival in Havasu Creek. Of variables with PCA loadings >0.4 or <−0.4 (the top 4) tested in separate models, rainbow trout ranked highest based on AIC_c_, followed by the timing of large flood post‐translocation, number of translocated chub; models were all within 2 ΔAIC_c_ of the top model, suggesting similar support.

Survival, *p* (pre‐flood), *R*, and *R′* varied for humpback chub translocated to Shinumo Creek (Figure 5); however, no covariates were retained in the top JLRR model (Table 3). Survival ranged from 0.56 to 0.99, with a sharp decline concurrent with the Galahad Fire and subsequent flooding in July 2014 (Figure 5). Models with *p =* 1, and fidelity = 0 during post‐fire recapture occasions would not converge, but we found the most support for time‐invariant fidelity differing before (0.85, 95% C.I. 0.82–0.87) and after (0.37, 95% C.I. 0.30–0.46) the 2014 fire and flood. Models with *F* ^*′*^ = 0 were ranked higher than those without constraints, supporting the assumption of high probability of emigration once individuals were detected at the PIA (Spurgeon, Paukert, Healy, Trammell, et al., 2015). With the exception of confounded or inestimable resight probability estimates for the last two intervals, *R* estimates were generally higher (range 0.03–0.12) than for Havasu Creek fish, and *p* ranged from 0.37 to 0.68 (Appendix S2: Figure S8).

### Havasu Creek recruitment and population growth

**FIGURE 6:**
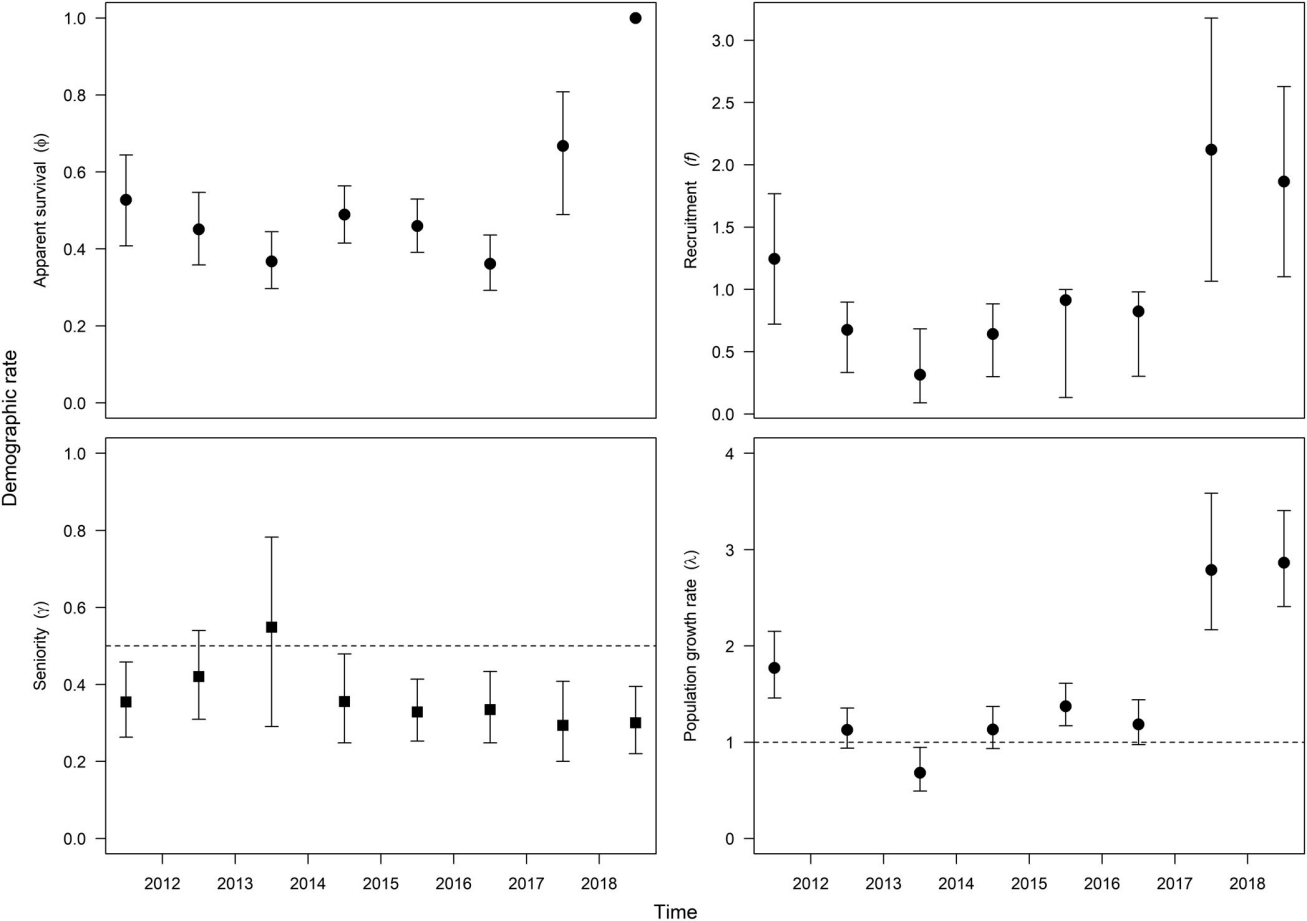
Havasu Creek temporal symmetry model results including apparent survival for all cohorts (translocated and non‐translocated fish), and seniority, recruitment, and population growth rate estimates for humpback chub produced *in situ* between 2011–2012 through 2018–2019. The 2018–2019 apparent survival estimate is confounded.

Temporal symmetry models with time‐varying annual apparent survival (φ) and recruitment (*f*), and without group effects on recapture probability (*p*_*j*_; mean 0.72, range 0.26–0.91), outperformed those with group‐specific parameters (*p*_*j*_; Table 3), which allowed us to leverage data from both groups (translocated and *in situ*‐produced fish) and estimate *f* rates for fish produced *in situ* (recruits), while testing recruitment hypotheses using covariates (adjusted for overdispersion, median $\hat{c}$ = 2.33, Table 3). Recruitment of the translocated group was directly related to translocations, and therefore, ignored. The greatest annual population growth rate (λ) of *in situ*‐produced humpback chub occurred in the last 2 years of our study in Havasu Creek, coinciding with the highest *f* rates (Figure 6). Population growth rates were <1 in the 2013–2014 interval, but were stable (λ 95% confidence intervals overlapped 1 in 3/8 intervals) or increasing (λ > 1, 4/8 intervals) in all other years. Of the 232 non‐translocated individuals captured and tagged, we observed the highest numbers of recruits in spring of 2018 (29) and 2019 (52). Both natal year humpback chub (coefficient = −0.84, SE = 0.43) and rainbow trout (coefficient = −0.32, SE = 0.16) abundance were retained in the top model (Akaike weight = 0.36, model likelihood = 1). There was also support for models that included natal year flood‐pulse frequency (ΔQAIC_c_ = 1.62; Akaike weight = 0.15, model likelihood = 0.44), in addition to humpback chub and rainbow trout abundance, and for a model without covariates on *f* (ΔQAIC_c_ = 1.88; Burnham & Anderson, 2002; Table 3). However, the SE for the flood‐pulse frequency covariate was large (coefficient = −0.64, SE = 1.01), and confidence intervals overlapped zero, suggesting a weak effect. Nonetheless, these results support density‐dependence hypotheses of reduced *f* with higher age‐1 or older humpback chub and invasive rainbow trout abundances during a cohort's natal year. In the top model, annual φ for all cohorts ranged 0.36–0.67, and our estimate of seniority (γ) indicated *f* was of greater importance to λ than φ in Havasu Creek in all years but two (i.e., *f* was proportionally more important than adult survival, γ < 0.5, and confidence intervals overlapped 0.5 in 2 years; Figure 6).

## DISCUSSION

Our study provides a rare example of robust demographic rate estimates and their relationships to intrinsic and extrinsic factors in small translocated populations of an imperiled fish. While translocations are often used for species recovery, very few are monitored effectively to allow for an assessment against predetermined objectives and adaptation of methodology (i.e., while accounting for detection probability; Nichols & Armstrong, 2012). The flow regime, often described as the “master variable” driving processes in streams, was important for somatic growth in both translocation sites, and positively related to survival, with the exception of a catastrophic flood event following a fire. Documentation of quantitative relationships between high flows and growth and survival of non‐salmonid fishes is limited (Rosenfeld, 2017). We also provide strong evidence supporting hypotheses of the negative impacts of invasive rainbow trout on humpback chub vital rates, and intraspecific density dependence in survival, growth, and recruitment. Our study is unique in that research in arid or semiarid watersheds with minimally impacted hydrologic regimes has been largely directed toward understanding patterns of persistence in native and introduced fishes in these dynamic systems (e.g., Propst & Gido, 2004; Stefferud et al., 2011), but few studies have directly addressed the potential mechanisms driving demographic rates, and analyses of translocations are rare.

Flooding can both limit and enhance the somatic growth rates of fishes (Arndt et al., 2002; Mallen‐Cooper & Stuart, 2003). We provide support for summer flooding as an important hypothesized seasonal driver of growth (Behn & Baxter, 2019; Dzul et al., 2016). Growth in humpback chub was constrained during winter, as expected for other warmwater fishes (Matthias et al., 2018; Pine et al., 2017), but we also observed a negative relationship between flooding and growth during fall–winter months in both translocated populations. Dzul et al. (2016) reported a similar negative relationship to winter–spring flooding related to snowmelt or winter rains and higher turbidity, because spring flooding may also alter temperatures and impact growth (Dzul et al., 2017). Compared with the LCR, winter floods are generally of much shorter duration in Havasu Creek, and scouring during these intense winter floods may temporarily remove periphyton or invertebrates. Subsequent production would be limited in winter relative to summer, due to reduced solar insolation (Hall et al., 2015). While we are not certain of the mechanism (e.g., increased terrestrial‐based allochthonous food delivery vs. instream autochthonous invertebrate production), our results support previous findings that food availability may be enhanced for desert river fishes during monsoon flooding (Behn & Baxter, 2019; Sabo et al., 2018), and importantly, opportunistic feeding on allochthonous matter translates into greater growth. The mechanism driving the positive response in growth rates to flooding in our perennially flowing systems is likely to differ from other arid‐land rivers where intermittent floods promote autochthonous production in floodplains and waterholes (Arthington & Balcombe, 2011). The importance of increased resource availability during floods for growth and recruitment is well documented for rivers with floodplains (Power et al., 1995, reviewed in Humphries et al., 2020), and for littoral‐dependent fishes in large rivers (Gutreuter et al., 1999), but less so in canyon‐bound streams such as those studied here (Behn & Baxter, 2019). Terrestrial‐based diet items may be critical to sustain drift‐foraging fishes in Havasu Creek (Garman, 1991; Kawaguchi et al., 2003), where instream invertebrate production is limited due to travertine deposition (Oberlin et al., 1999; Rundio, 2009). Monsoon flood‐pulsed food in Havasu Creek may offset intraspecific, density‐dependent negative effects on growth. Nonetheless, we suspect enhanced food availability in our sites would be short‐lived, given the intensity and short duration of monsoon flood events. Our results suggest the physiological capacity of humpback chub to process food evolved for boom and bust cycles (Armstrong & Schindler, 2011), which warrants further study.

From a bioenergetic standpoint, consumption and demand for food, as well as intraspecific and interspecific competition, would be higher during warmer summer periods (Paukert & Petersen, 2007; Taniguchi et al., 1998). Temperature and food availability do in fact interact to influence growth of humpback chub in the LCR (Dzul et al., 2017), and in other species (reviewed in Ficke et al., 2007, e.g., Pennock et al., 2020). Rainbow trout are also known to aggressively defend foraging territories in streams (Keeley, 2001), potentially to the detriment of humpback chub growth and survival (Yackulic et al., 2018). Therefore, bioenergetic interactions, which are driven in part by temperature, may explain the importance of the interactions of rainbow trout, season, and flooding on growth in Shinumo Creek, where substantial diet overlap was documented between the two species (Spurgeon, Paukert, Healy, Kelley, et al., 2015). Because growth rate–body size relationships are linked to survival and adult fecundity, understanding drivers of growth at early life stages that may manifest in the fitness of adult fishes (Nater et al., 2018; Vincenzi, Satterthwaite, et al., 2012) may be critical to the success of translocations. Faster growth may manifest in gape‐limited predator avoidance (Urban, 2007), earlier age‐at‐maturity (Stone et al., 2020), and increased fecundity, which would allow populations to recover quickly from losses due to predation or disturbance (Vincenzi, Satterthwaite, et al., 2012).

We identified functional relationships between annual humpback chub recruitment and age‐1 and older humpback chub (i.e., density dependent) and natal year rainbow trout abundances, and to a lesser extent, flood frequency, in Havasu Creek. The largest year classes of humpback chub recruited to the population in 2018 and 2019 (at age‐2). These fish would have been produced *in situ* in 2016 and 2017 natal years, following cessation of translocations, declines in humpback chub, and when few trout were present. The occurrence of intraspecific density‐dependent recruitment in fishes is commonly reported (Minto et al., 2008), but nonetheless controversial and potentially overridden by environmental conditions (Lobón‐Cerviá, 2014; Rose et al., 2001). Surprisingly, given the extremes in discharge observed during our study, flooding during the natal summer received less support in our recruitment models relative to hypothesized predation by rainbow trout (Coggins et al., 2011; Yard et al., 2011) or older conspecifics (Stone & Gorman, 2006). Nonetheless, monsoon flood magnitude during the natal year for the two largest cohorts ranked among the lowest (<14.3 m^3^/s, median max. Monsoon flood = 29.6 m^3^/s). High recruitment rates following years without intense monsoon floods suggests both flood magnitude and frequency may constrain recruitment (Healy, Schelly, et al., 2020). In contrast, intense monsoon flooding may result in gains in recruitment in intermittent river systems flowing through arid lands (Arthington & Balcombe, 2011). Although we lacked data to test the relationship between the timing of flooding and recruitment (emergence timing is unknown in Havasu Creek), monsoon flooding may cause dispersal of YOY humpback chub (Yackulic et al., 2014). Dispersal of larval fishes through flooding may be an important adaptive mechanism for recruitment in systems with patchy distribution of resources (e.g., food; Humphries et al., 2020; Winemiller & Rose, 1992). Flooding prior to spawning is also important for recruitment and persistence of stream fishes (Budy et al., 2015; Healy, Schelly, et al., 2020). Floods maintain channel complexity and create aerated substrates for lithophilic spawners including salmonids (Bestgen et al., 2020) and humpback chub (Gorman & Stone, 1999; van Haverbeke et al., 2013).

Our results suggest that invasive salmonids impacted recruitment and growth in Havasu Creek, as found for humpback chub in the Colorado River (Coggins et al., 2011; Yackulic et al., 2018). The likely mechanism explaining the relationship between recruitment and rainbow trout in Havasu Creek is related to rainbow trout predation upon juvenile humpback chub (Coggins et al., 2011, Yard et al., 2011). Rainbow trout are one of a suite of globally introduced (Crawford & Muir, 2008) invasive salmonids implicated in the suppression of native fish recruitment through piscivory (McDowall, 2006; e.g., New Zealand, Jellyman & Mcintosh, 2010; South Africa, Shelton et al., 2015) and other multilevel ecological impacts (Hansen et al., 2019; McIntosh et al., 2011; Simon & Townsend, 2003; Stanković et al., 2015). While we can only speculate on the cause for a lack of recruitment in Shinumo Creek prior to extirpation, rainbow trout predation on larval chub is one hypothesis. Whiting et al. (2014) demonstrated that rainbow trout could have a substantial impact on a small‐bodied native fish population, and Spurgeon, Paukert, Healy, Kelley, et al. (2015) found 75% of large rainbow trout stomachs to contain native fish in Shinumo Creek. The highest incidence of piscivory corresponded to June, when native fishes would be at their highest abundances following spawning (Spurgeon, Paukert, Healy, Kelley, et al., 2015). The discovery of juvenile native suckers and large increases in native fish abundance (~480%) following the suppression of rainbow trout and brown trout (*Salmo trutta*) in another GCNP tributary also lends support to this hypothesis (Healy, Schelly, et al., 2020). Nonetheless, other authors have suggested the effects of warming temperatures in the thermally altered Colorado River may override or lessen trout predation risks to juvenile humpback chub (Coggins et al., 2011; Ward & Morton‐Starner, 2015; Yackulic et al., 2018). Our findings appear contrary, because our study was conducted in naturally warmer and more variable thermal regimes than in the Colorado River; temperatures only rarely dropped below the approximate minimum threshold for growth in Havasu Creek, for example. Projections suggest the consequences of basin‐wide water storage decisions may override climate change in governing future Colorado River temperatures (Dibble et al., 2021). Future water management decisions that consider the impacts to endangered fish could be informed by additional knowledge of the interactions between rainbow trout and humpback chub across a broader temperature range than in previous laboratory (10–20°C, Ward & Morton‐Starner, 2015) or field studies (<15°C, Yackulic et al., 2018). Our results provide further support for the eradication of invasive species to facilitate the successful reintroduction or recovery of animal populations (e.g., salmonids, Al‐Chokhachy et al., 2009; amphibians, Bosch et al., 2019).

We found evidence of intraspecific density dependence in survival, moderated by flooding, in humpback chub translocated to Havasu Creek. The relationships between survival and humpback chub abundance based on catch, the number of humpback chub translocated, and the timing of a large flood event in relationship to translocation timing – all correlated variables represented on Havasu Creek PC2 in our best‐supported models – provided evidence for the density‐dependence survival hypotheses. Survival was lowest during the summer intervals with the highest total number of humpback chub present (2014–2016). The discovery of density dependence in vital rates has important implications for management of stocked or translocated populations because densities are being directly manipulated (Lorenzen & Enberg, 2002). Reintroducing or augmenting populations with numbers that exceed the carrying capacity would therefore be counterproductive. However, detection of density dependence in subadult or adult life stages, and understanding how population dynamics are influenced can be difficult (reviewed in Rose et al., 2001). Results of post hoc tests suggest complex and confounding relationships that confuse the interpretation of mechanistic survival relationships. For example, survival was positively, albeit less strongly (i.e., relatively weakly related to PC2), related to rainbow trout abundance. Reduced body condition found following intervals with greater humpback chub abundance (B. Healy, *unpublished relative weight data*), and negative relationships between humpback chub abundance and individual growth rates in this study, provide additional lines of evidence supporting the density‐dependence hypotheses. Declining individual growth rates and body condition are linked to lower survival in fishes (Korman et al., 2021). Evidence for density‐dependent survival has also been noted in the LCR population, but generally limited to juveniles (Pine et al., 2013; van Haverbeke et al., 2013; Yackulic et al., 2018), as is common in other fishes (Lobón‐Cerviá, 2012 may be an exception; Vincenzi et al., 2016).

High mortality in humpback chub translocated to Shinumo Creek appeared to coincide with intense, ash‐laden flooding. Despite the lack of covariates in our best Shinumo Creek survival models, we observed a sharp decline in interval‐specific survival coinciding with the 2014 flood event, confirming high mortality predictions, rather than emigration from the Creek. In contrast, we observed relatively weak but positive relationships between survival and flooding in Havasu Creek, which is notable because extreme floods (i.e., more than two orders of magnitude above baseflow) occurred in half the years, and sometimes multiple times within a year. The absence of a catastrophic effect of extreme flooding, or even a beneficial effect, suggests high resistance to flooding of subadult and older humpback chub in Havasu Creek. High resistance and resilience to flooding would be consistent with findings for native fishes in other arid‐land systems (Pearsons et al., 1992; Propst et al., 2008; Rogosch et al., 2019). In contrast, ash‐laden floods commonly extirpate aquatic biota in receiving waters due to hypoxia or toxic water chemistry (Bixby et al., 2015; Whitney et al., 2015). The extirpation of the Shinumo Creek population, as well as native resident bluehead sucker, suggests fire‐related flood events – the type of event projected to increase in frequency under some future climate scenarios (O'Donnell et al., 2018) – could lead to potential peril for small translocated populations.

Surprisingly, flow‐related covariates were unimportant in explaining variation in fidelity in both translocation sites, despite high emigration rates found by Spurgeon, Paukert, Healy, Trammell, et al. (2015) associated with higher stream stage in Shinumo Creek. We also noted much lower fidelity rates during intervals corresponding with the largest monsoon‐driven Havasu Creek flood events during our study, occurring in the summers of 2013 and 2018. Covariates representing humpback chub abundance and the timing of large Havasu Creek flood events (>28 m^3^/s) following translocations were corelated on PC2. This pattern simply suggests longer time periods between translocations and the occurrence of large floods led to higher numbers of humpback chub. We would expect newly released fish having been reared in a hatchery for up to a year to fare poorly in the face of a large disturbance or other stressful event. However, once established, native fishes appear to resist high flow events through morphological, physiological, or behavioral adaptations that may prove advantageous over invasive species (Moran et al., 2018; Ward et al., 2003).

Our ability to infer relationships between vital rates and abiotic and biotic drivers benefited from a biannual mark–recapture monitoring regime (sensu Wheeler et al., 2018) designed to answer questions related to the translocations developed a priori (e.g., Trammell et al., 2012); a practice uncommon in many reintroduction programs (Armstrong & Seddon, 2008; Nichols & Williams, 2006). Although our findings related to drivers of recruitment are supported in the literature (e.g., negative effects of flooding and invasive trout) as described above, we suggest additional years (~10 or more) of monitoring will allow for differentiation between sampling and process variation, and in turn, stronger inference (Burnham & White, 2002). High emigration immediately after release (35% within 25 days, Spurgeon, Paukert, Healy, Trammell, et al., 2015), and short residence time prior to extirpation probably limited our ability to estimate fidelity and test hypotheses for Shinumo Creek humpback chub. In prior analyses, we observed that survival was related to size at release; however, survival rate estimation was confounded by emigration (Healy, Omana Smith, et al., 2020; Spurgeon, Paukert, Healy, Trammell, et al., 2015), which may be influenced by fish size or age (Yackulic et al., 2014). We leveraged detection data from multiple monitoring programs throughout the CRE allowing for improved survival estimates accounting for emigration. These survival estimates (annual mean survival = 0.60 and 0.35, Havasu and Shinumo, respectively) were comparable with those found for juvenile humpback chub in the source population (Dzul et al., 2016; Yackulic et al., 2014), and slightly lower, for Shinumo, to fish translocated to the upper LCR (Yackulic et al., 2021). We also estimated lower survival for *in situ*‐produced fish, which could have been a function of unaccounted for tag‐loss in the field. Alternatively, higher survival of translocated fish shows the collection and rearing process was advantageous, and suggests mortality related to the stress of transport to release sites and handling during the tempering and release process were unimportant (Tennant et al., 2019) relative to natural conditions. Future work could involve investigating the value of translocations to tributaries outside the LCR using vital rates generated by our study, in a cost–benefit framework relative to other conservation actions (Lamothe et al., 2021; Yackulic et al., 2021).

### Implications for conservation

Our results provide further evidence for demographic resilience (Capdevila et al., 2020) of arid‐land fishes adapted to relatively frequent flood disturbances (Eby et al., 2003; Stefferud et al., 2011). Three of eight annual population growth rate estimates were high (λ > 1.5) for humpback chub, suggesting the potential for rapid recovery under ideal conditions, such as following summers lacking intense monsoon flooding. The continued presence of invasive fishes may nonetheless limit demographic responses and reduce resiliency. Vincenzi et al. (2016) documented similar demographic responses to disturbance for populations of an imperiled salmonid, but resiliency to ash‐laden flooding in arid‐land streams may depend on the spatial location of disturbances and connectivity to sources for recolonization (Gido et al., 2019). Establishing populations with connections to broader stream networks would ensure population persistence. Smaller isolated tributary populations may take longer to recover to pre‐disturbance levels than those with more direct connections to source populations in a mainstem river (Gido et al., 2019). Continued monitoring would be necessary to understand how humpback chub demographic rates in translocated populations ultimately translate to long‐term persistence.

The relationships between demographic rates and stream flow patterns we observed have important implications for conservation under climate change. The region is projected to become drier with increasing wildfire severity (O'Donnell et al., 2018) that could lead to more frequent ash‐laden floods, and declining baseflows or spring flooding may limit humpback chub production (van Haverbeke et al., 2013). If maintaining tributary populations in the fragmented CRE is a goal, occasional augmentation following disturbances, and focused mitigation of limiting factors, including removal of invasive species may be necessary. Regardless, the existence of density dependence in vital rates reinforces the importance of the existing population size, carrying capacity, and invasive species densities when planning augmentation and translocations programs. Reductions in spring flood magnitude and declining baseflow under extended drought scenarios projected for spring‐fed tributaries (Tillman et al., 2020) would probably further constrain carrying capacities in our sites and others in arid‐land systems. Warming temperatures with declining tributary baseflows (Bair et al., 2019) will also intensify consumptive demand and potentially increase competition for food between rainbow trout and humpback chub. Additional study is needed to understand how tributary flow and thermal regimes may change in future years, and how these novel regimes may mediate biotic interactions among native and introduced fishes. Despite these uncertainties, our findings derived from monitoring outcomes against a priori defined objectives can provide the basis for future adaptive management of translocated populations (Runge, 2011; Runge et al., 2011).

In contrast with predictions of life history models suggesting a lack of density dependence in recruitment (“periodic strategist,” Winemiller, 2005), we found population growth rates in endangered and long‐lived humpback chub were driven primarily by density‐dependent reproduction and recruitment in the early years of life. Studies finding density dependence in recruitment are generally less common than those identifying density‐dependent somatic growth in fishes (Grossman & Simon, 2019). Adult survival was a less important component contributing to annual population growth rates of humpback chub in Havasu Creek compared with recruitment (all but 2 years), indicating management regimes aimed at mitigating factors limiting recruitment would lead to population maintenance or growth (Coggins et al., 2006). Protecting natural flow regimes in the Grand Canyon region will allow for continued pulses of food to both tributaries and the mainstem (Sabo et al., 2018). Our work also supports the need to achieve suppression of invasive fishes prior to translocations (Al‐Chokhachy et al., 2009; Cochran‐Biederman et al., 2015).

In conclusion, we demonstrate how translocations can provide unique opportunities to study ecological processes. With thorough monitoring and detailed analyses, we provided additional knowledge of the life history and drivers of population dynamics of an imperiled species that can assist in planning of recovery actions, and inform further hypothesis testing through the use of models (Armstrong & Reynolds, 2012; Sarrazin & Barbault, 1996). We also improved our knowledge of basic humpback chub ecology and interactions of this endangered species with an introduced species and its environment (Sarrazin & Barbault, 1996). Our study presents a rare example of a successful reintroduction effort of an endangered species, while also elucidating factors preventing successful recruitment, and ultimate extirpation, of another translocated population; both cases will inform future actions aimed at stemming global‐scale biodiversity loss (Tickner et al., 2020).

## CONFLICT OF INTEREST

The authors declare no conflict of interest.

## Supporting information


Appendix S1

Appendix S2

## Data Availability

Data (Healy et al., 2022) are available in Figshare at https://doi.org/10.6084/m9.figshare.c.5805593.v1.
