# Supplementary material for: Life and death in a dynamic environment: Invasive trout, floods, and intraspecific drivers of translocated populations
Source: Ecol Appl. 2022 Jun 13;32(6):e2635. doi: 10.1002/eap.2635 (PMC9541007; doi:10.1002/eap.2635)
Supplement: Supplementary file 1 — Appendix S1 [file EAP-32-e2635-s002.pdf]

Supporting Information. Brian D. Healy, Phaedra Budy, Mary M. Conner, Emily C. Omana Smith. 2022. Life and death in a dynamic environment: Invasive trout, floods, and intra-specific drivers of translocated populations. *Ecological Applications.*

Appendix S1. Study species and hypotheses

What is known of the population ecology and life history of humpback chub is based on studies in the LCR and adjacent Colorado River in Grand Canyon, which began in the 1980s (Kaeding and Zimmerman 1983, Valdez and Ryel 1995, Coggins et al. 2006, Yackulic et al. 2014). As expected for a long-lived (>30 years) organism, long-term mark-recapture studies have shown adult survival to be high (up to 82% annual survival, Coggins et al. 2006), but variable depending on primary residency in the LCR or Colorado Rivers (61% vs. 78%, Yackulic et al. 2014). Juvenile and young-of-year (YOY) survival can vary dramatically between years, and was thought to be particularly low for fish swept out of the LCR and into the colder Colorado River during monsoon flooding (Valdez and Ryel 1995, Robinson and Childs 2001). Cold hypolimnetic discharge from Glen Canyon Dam favors introduced salmonids, which compete with and prey upon humpback chub (Yard et al. 2011), leading to reduced growth and survival in the LCR inflow reach (Coggins et al. 2011, Yackulic et al. 2018). Yackulic et al. (2014) found high emigration rates of LCR juveniles during monsoon season (July to September) and survival of these emigrants near the Colorado-LCR confluence varied with the abundance of rainbow trout *Oncorhynchus mykiss* (Yackulic et al. 2018). Warmwater invasive fishes (Marsh and Douglas 1997), or introduced parasites (Campbell et al. 2019) may also threaten humpback chub in the LCR, where survival of all age-classes was surprisingly lower relative to the mainstem (Yackulic et al. 2014). Differences in growth of humpback chub in the LCR and mainstem has been largely attributed to thermal regime differences, whereas the LCR is >6°C warmer

(Yackulic et al. 2014), but variation in growth within the LCR may be driven by food availability as well as temperature (Dzul et al. 2017, Stone et al. 2020), and winter flooding may limit growth (Dzul et al. 2016).

Managers were prompted to consider means to establish new spawning populations in tributaries with more benign conditions, including through translocations, following decadal-scale declines in abundance of humpback chub (Coggins et al. 2006), persistent threats as described above, and the reliance of the population on reproduction in only the LCR (Valdez et al. 2000). Translocations of humpback chub were first initiated to vacant upstream reaches of the LCR in 2003, where fish remained and grew rapidly (Stone et al. 2020). Building on successes in the LCR, we initiated translocations to Shinumo (2009 - 2013, Spurgeon et al. 2015b) and Havasu Creeks (2011 - 2016, Trammell et al. 2012, Healy et al. 2020a; Table S1). Tributaries targeted for translocations are much smaller than others supporting humpback chub populations, but were thought to be suitable to support small populations with fewer threats from invasive fishes (Valdez et al. 2000, Pine et al. 2013).

Survival and growth rates in translocated populations in Shinumo and Havasu Creeks were estimated in two prior studies using mark-recapture methods (Spurgeon et al. 2015b, Healy et al. 2020a). While apparent survival (survival confounded by emigration) and individual growth rates in translocated populations were comparable to those of juvenile humpback chub in the LCR, neither study assessed environmental drivers of these vital rates, and study designs were inadequate to estimate true survival. Further, as determined through detections on a passive-integrated transponder (PIT) tag antenna array, nearly half of translocated individuals left in Shinumo Creek within the first year, associated with increasing flow and temperature (Spurgeon et al. 2015b), which potentially limiting the establishment of the population (Pine et

al. 2013). The remaining individuals were extirpated from Shinumo Creek during July – August of 2014, following a series of large flood events triggered by intense rainstorms on a fire. In contrast, we observed reproduction and recruitment in the Havasu Creek population, which persists through 2020. Survival of emigrants and fidelity rates are unknown for translocated cohorts due to imperfect detection, and our study aims to quantify these rates, which would not be possible without the inclusion of detections outside release sites (Barker 1997, Schaub and Royle 2014). Through monitoring conducted by cooperators throughout the Colorado River Ecosystem (CRE; US Geological Survey -Grand Canyon Monitoring and Research Center (GCMRC), unpublished data), defined as the Colorado River and its tributaries in Grand Canyon, and through our own monitoring in the Colorado River adjacent to Shinumo Creek, we have detected individuals that had emigrated from both translocation sites.

Based on these prior studies, we assessed evidence for the following hypothesized relationships between flow, thermal characteristics, and invasive salmonids, and translocated humpback chub population dynamics and individual growth:

1) Individual growth, and recruitment and survival rates will vary with flood frequency, magnitude, timing, and duration. Growth of subadults would be higher during summer months in years with higher frequency of floods that would deliver additional terrestrial diet items (Behn and Baxter 2019) or scour substrates to enhance invertebrate growth – of particular importance in Havasu Creek where invertebrate production appears limited relative to other tributaries (Oberlin et al. 1999; Figure S1). While gut fullness in humpback chub was found to be highest during periods of flooding in the LCR (Behn and Baxter 2019), it is uncertain whether the addition of allochthonous food items would translate into higher growth rates. Terrestrial-derived food quality may vary (Brett et al. 2017), and in one translocated population of humpback chub,

assimilation of allochthonous diet items was low relative to others (e.g., fish, insects, algae; Spurgeon et al. 2015a). Winter flooding may also limit growth, as in the LCR (Dzul et al. 2016).

We predicted recruitment would be limited during years with higher monsoon flood frequency or intensity, as in the LCR where YOY are transported downstream to the Colorado River (Yackulic et al. 2014). Unlike in the LCR, flood-dispersed juveniles are unable to return to and recruit into translocated populations due to barrier falls near the mouth of each tributary. Once recruited into the sub-adult or adult population, we would expect minimal effects of flooding on survival, with the exception of ash-laden floods following fires in the Shinumo Creek watershed. Southwestern native fishes vary in their resistance to ash and intense flooding, and a congener was susceptible to ash flows in another Colorado River tributary system (Gido et al. 2019). It is unclear if humpback chub were flushed from Shinumo Creek or suffered high mortality rates during the summer of 2014, but we suspected higher mortality rates occurred.

2) The strength of density-dependence in vital rates was assumed to be greater for juveniles, and weaken with size and age in the LCR by Pine et al. (2013), but Yackulic et al. (2018) found only weak support for density-dependent growth and survival in humpback chub in the Colorado River. Nonetheless, following the onset of reproduction, increasing annual abundance estimates of Havasu Creek humpback chub began to level off, and somatic growth rates were lowest in the largest cohort translocated (2014), suggesting density dependence (Healy et al. 2020a). Therefore, we expect density-dependent growth and recruitment in Havasu Creek, but relationships between density and vital rates may be less important in Shinumo Creek compared to other drivers, given high emigration rates (Spurgeon et al. 2015b), and more abundant food (Figure S1; NPS unpublished data).

3) We hypothesize rainbow trout would limit growth, survival, and ultimately recruitment in translocation sites. Previous food web analysis showed high trophic niche overlap in Shinumo Creek between rainbow trout and humpback chub (Spurgeon et al. 2015a), suggesting potential competition for food. Higher rates of direct predation by rainbow trout upon YOY or sub-adult humpback chub (cf. Yard et al. 2011) would limit recruitment and survival in years when trout are abundant (Yackulic et al. 2018). Alternatively, trout densities appear to be low based on field observations and catch rates through the duration of our study in Havasu (also see apendix in Healy et al. 2020a) relative to Shinumo Creek, and thus trout may have minimal impact on vital rates in Havasu Creek. While no humpback chub were recovered from rainbow trout stomachs during monitoring in our translocation sites, bite scars were observed, piscivory upon other (more abundant) resident native fishes occurred (Whiting et al. 2014, Spurgeon et al. 2015a), and trout were found to suppress native cyprinid and catostomid distribution and abundance in another tributary (Healy et al. 2020b), suggesting the potential for negative interactions between the two species.

LITERATURE CITED

Barker, R. J. 1997. Joint modeling of live-recapture , tag-resight , and tag-recovery data. Biometrics 53:666–677.

Behn, K. E., and C. V. Baxter. 2019. The trophic ecology of a desert river fish assemblage: influence of season and hydrologic variability. Ecosphere 10:1–24.

Brett, M. T., S. E. Bunn, S. Chandra, A. W. E. Galloway, F. Guo, M. J. Kainz, P. Kankaala, D. C. P. Lau, T. P. Moulton, M. E. Power, J. B. Rasmussen, S. J. Taipale, J. H. Thorp, and J. D. Wehr. 2017. How important are terrestrial organic carbon inputs for secondary production in freshwater ecosystems? Freshwater Biology 62:833–853.

Campbell, M. C., C. A. Caldwell, T. D. Lewis, W. D. Wilson, and C. C. Gard. 2019. Nonlethal detection of Asian fish tapeworm in the federally endangered Humpback Chub using a molecular screening tool. Transactions of the American Fisheries Society 148:832–842.

Coggins, L. G., W. E. Pine, C. J. Walters, D. R. Van Haverbeke, D. Ward, and H. C. Johnstone. 2006. Abundance trends and status of the Little Colorado River population of humpback chub. North American Journal of Fisheries Management 26:233–245.

Coggins, L. G., M. D. Yard, and W. E. Pine. 2011. Nonnative fish control in the Colorado River in Grand Canyon, Arizona: an effective program or serendipitous timing? Transactions of the American Fisheries Society 140:456–470.

Dzul, M. C., C. B. Yackulic, J. Korman, M. D. Yard, and J. D. Muehlbauer. 2017. Incorporating temporal heterogeneity in environmental conditions into a somatic growth model. Canadian Journal of Fisheries and Aquatic Sciences 74:316–326.

Dzul, M. C., C. B. Yackulic, D. M. Stone, and D. R. Van Haverbeke. 2016. Survival, growth, and movement of subadult humpback chub, Gila Cypha, in the Little Colorado River, Arizona. River Research and Applications 32:373–382.

Gido, K. B., D. L. Propst, J. E. Whitney, S. C. Hedden, T. F. Turner, and T. J. Pilger. 2019. Pockets of resistance: response of arid-land fish communities to climate, hydrology, and wildfire. Freshwater Biology:1–17.

Healy, B. D., E. C. Omana Smith, R. C. Schelly, M. A. Trammell, and C. B. Nelson. 2020a. Establishment of a reproducing population of endangered humpback chub through translocations to a Colorado River tributary in Grand Canyon, Arizona. North American Journal of Fisheries Management 40:278–292.

Healy, B. D., R. C. Schelly, C. B. Yackulic, E. C. O. Smith, and P. Budy. 2020b. Remarkable

response of native fishes to invasive trout suppression varies with trout density, temperature, and annual hydrology. Canadian Journal of Fisheries and Aquatic Sciences 77:1446–1462.

Kaeding, L. R., and M. A. Zimmerman. 1983. Life history and ecology of the humpback chub in the Little Colorado and Colorado Rivers of the Grand Canyon. Transactions of the American Fisheries Society 112:577–594.

Marsh, P. C., and M. E. Douglas. 1997. Predation by introduced fishes on endangered humpback chub and other native species in the Little Colorado River, Arizona. Transactions of the American Fisheries Society 126:343–346.

Oberlin, G. E., J. P. Shannon, and D. W. Blinn. 1999. Watershed influence on the macroinvertebrate fauna of ten major tributaries of the Colorado River through Grand Canyon , Arizona. The Southwest Naturalist 44:17–30.

Pine, W. E., B. Healy, E. O. Smith, M. Trammell, D. Speas, R. Valdez, M. Yard, C. Walters, R. Ahrens, R. Vanhaverbeke, D. Stone, and W. Wilson. 2013. An individual-based model for population viability analysis of humpback chub in Grand Canyon. North American Journal of Fisheries Management 33:626–641.

Robinson, A. T., and M. R. Childs. 2001. Juvenile growth of native fishes in the Little Colorado River and in a thermally modified portion of the Colorado River. North American Journal of Fisheries Management 21:809–815.

Schaub, M., and J. A. Royle. 2014. Estimating true instead of apparent survival using spatial Cormack-Jolly-Seber models. Methods in Ecology and Evolution 5:1316–1326.

Spurgeon, J. J., C. P. Paukert, B. D. . Healy, C. A. Kelley, and D. P. Whiting. 2015a. Can translocated native fishes retain their trophic niche when confronted with a resident

invasive? Ecology of Freshwater Fish 24:456–466.

Spurgeon, J. J., C. P. Paukert, B. D. Healy, M. Trammell, D. Speas, and E. Omana Smith. 2015b. Translocation of humpback chub into tributary streams of the Colorado River: implications for conservation of large-river fishes. Transactions of the American Fisheries Society 144:502–514.

Stone, D. M., M. J. Pillow, K. L. Young, D. R. Van Haverbeke, and J. D. Walters. 2020. Effects of Disparate Water Temperatures and Food Bases on Humpback Chub Growth Rates within the Little Colorado River , Arizona. North American Journal of Fisheries Management:1–13.

Trammell, M., B. D. Healy, E. O. Smith, and P. Sponholtz. 2012. Humpback chub translocation to Havasu Creek , Grand Canyon National Park : implementation and monitoring plan. Fort Collins, Colorado.

Valdez, R. A., M. E. Douglas, M. Douglas, R. J. Ryel, K. R. Bestgen, and D. L. Wegner. 2000. Research and implementation plan for establishing a second population of humpback chub in Grand Canyon. Grand Canyon Monitoring and Research Center, U. S. Department of Interior. Flagstaff, Arizona.

Valdez, R. A., and R. J. Ryel. 1995. Life history and ecology of the humpback chub (Gila cypha) in the Colorado River, Grand Canyon, Arizona. Logan, Utah.

Whiting, D. P., C. P. Paukert, B. D. Healy, and J. J. Spurgeon. 2014. Macroinvertebrate prey availability and food web dynamics of nonnative trout in a Colorado River tributary, Grand Canyon. Freshwater Science 33:872–884.

Yackulic, C. B., J. Korman, M. D. Yard, and M. Dzul. 2018. Inferring species interactions through joint mark–recapture analysis. Ecology 99:812–821.

Yackulic, C. B., M. D. Yard, J. Korman, and D. R. Van Haverbeke. 2014. A quantitative life history of endangered humpback chub that spawn in the Little Colorado River: Variation in movement, growth, and survival. Ecology and Evolution 4:1006–1018.

Yard, M. D., L. G. Coggins, C. V. Baxter, G. E. Bennett, and J. Korman. 2011. Trout piscivory in the Colorado River, Grand Canyon: effects of turbidity, temperature, and fish prey availability. Transactions of the American Fisheries Society 140:471–486.
