# Supplementary material for: Life and death in a dynamic environment: Invasive trout, floods, and intraspecific drivers of translocated populations
Source: Ecol Appl. 2022 Jun 13;32(6):e2635. doi: 10.1002/eap.2635 (PMC9541007; doi:10.1002/eap.2635)
Supplement: Supplementary file 2 — Appendix S2 [file EAP-32-e2635-s001.pdf]

Supporting Information. Brian D. Healy, Phaedra Budy, Mary M. Conner, Emily C. Omana Smith. 2022. Life and death in a dynamic environment: Invasive trout, floods, and intra-specific drivers of translocated populations. *Ecological Applications.*

Appendix S2. Somatic growth and mark-recapture modeling

Modeling drivers of individual growth – We used linear mixed-effects models (Gelman and Hill 2009, Weisberg et al. 2010, Dzul et al. 2017) to evaluate combinations of predictors of individual somatic growth rates for summer and winter seasons of the first year following translocation of each cohort of humpback chub. We calculated individual growth rates for the 2013 Shinumo Creek cohort using the formula: growth $_{season}$ = $TL_{time-2} – TL_{time-1}/\Delta$-day (Spurgeon et al. 2015, Healy et al. 2020), to maintain consistency with published humpback chub growth rates in Shinumo Creek from 2009 – 2011 (Spurgeon et al. 2015), and those for juvenile humpback chub translocated to Havasu Creek between 2011 – 2014 in Healy et al. (2020), minus the 2013 Havasu Creek cohort (no data due to the fall 2013 U.S. government shutdown). To avoid potential autocorrelation related to repeated measures of PIT-tagged individuals and assess the strength of temporally-variable environmental or biological fixed effects in predicting growth rates, we included a random intercept representing individual humpback chub in all models (Weisberg et al. 2010). We attempted to include a random intercept representing translocated cohort membership, and a random slope interaction with fish size, however, our data did not always support this more complex random effects structure. Instead, we included only a random intercept representing variation that may be introduced related to the translocation year. We also included individual fish size measured prior to release (total length) as a fixed effect in all models to account for declining growth rates with size (Pine et al. 2017b):

$$y_{s,t} = \beta_0 + \beta_1 TL + \sum_{\boldsymbol{k}} \boldsymbol{\beta_k} \times \boldsymbol{Z_{k,s,t}} + \xi_{0[\boldsymbol{i}]} + \theta_{0[\boldsymbol{t}]} + \varepsilon_{s,t} \quad \text{(S1)}$$

$$\xi_0 \sim N(0, \sigma_i^2)$$

$$\theta_0 \sim N(0, \sigma_t^2)$$

$$\varepsilon_{s,t} \sim N(0,\ \sigma^2)$$

where $y_{s,t}$ represents the modeled growth rates of humpback chub during season *s* and year *t* (in Havasu or Shinumo creeks or both), β_0 is the intercept, $\beta_1 TL$ is the term representing humpback chub total lengths included in all models as a fixed effect, $\boldsymbol{Z}$ denotes the value(s) of environmental covariates $\boldsymbol{k}$ measured in intervals *s* of *t* and $\boldsymbol{\beta}$ represents values of fixed effects coefficients. Random intercepts for individual humpback chub (*i*) released ($\xi_{0[\boldsymbol{i}]}$), and each year $\theta_{0[\boldsymbol{t}],}$ are also included, and as for the model error term, $\varepsilon_{s,t}$, assumed to have a normal distribution (*N*), with a mean of 0 and standard deviation of σ^2, σ_i^2 and σ_t^2.

We tested for effects of between- and within-stream temporal variation in temperature, flood-pulse frequency, and density-dependence on growth rates using combinations of covariates (Table 1) for models incorporating all cohorts from both streams. In addition, we separately evaluated the relationship between rainbow trout and humpback chub growth rates, along with other covariates, within Havasu and Shinumo Creeks. To assess the potential for intra-specific density-dependent growth, and growth relationships with trout, we included abundance indices from the monitoring event at the end of each seasonal interval as a covariate. Our base model, and all others tested, included temperature, or a variable representing season and stream as factors, except where collinearity was introduced with the inclusion of season and other

predictors as explained below. In addition to additive models, we included interactions between flood pulse frequency and season, as well as between the humpback chub and rainbow trout abundance and flood-pulse frequency and season in candidate models. We minimized collinearity among our covariates by carefully considering the inclusion of predictors together in models with Pearson's (*r*) correlation coefficients >0.60 (see diagnostic process below), and replaced variables with *r* >0.70 (Zuur et al. 2010, Dormann et al. 2013). In the full dataset used to analyze predictors of growth in both streams, humpback chub abundance was correlated with temperature (*r* = 0.73) and rainbow trout (*r* = 0.76). High correlations were found among some Shinumo Creek covariates including between humpback chub abundance and temperature (*r* = 0.86), and between season and rainbow trout abundance (*r* = 0.69), and season and temperature (*r* = 0.70). Similarly, in the Havasu Creek dataset, the humpback chub abundance index was correlated with rainbow trout (*r* = 0.74), and flood-pulse frequency was correlated with both season (*r* = 0.69) and temperature (*r* = 0.77). In cases where collinearity (*r*>0.70) was evident, we substituted another ecologically-similar variable which we assumed to represent the hypothesized environmental or biological driver of interest. We included season in lieu of temperature, and avoided including both humpback chub and rainbow trout abundance in the same model. We reasoned that the season covariate would represent wide variation in seasonal temperature known to influence growth in humpback chub (Pine et al. 2017a), which is a necessary effect for inclusion in models, while also eliminating multiple correlations between variables (temperature and flood-pulse frequency, rainbow trout abundance, humpback chub abundance).

Growth model selection and diagnostics – Predictors were z-scored to aid in interpretation of partial regression coefficients (Gelman and Hill 2009). We constructed all

growth models with combinations of covariates using the lme4 package in R (Bates et al. 2015, R Core Team 2019), ranked models using AIC*c* (Burnham and Anderson 2002) calculated with the bblme package (Bolker and Team 2017), and used R^2 calculated for the fixed effects in the models using the sjplot package (Lüdecke 2019) for model selection and comparison.

Following model selection, we calculated the variance inflation factor (VIF) for each of our top ranked models using the car package in R (Fox and Weisberg 2014, R Core Team 2019), and carefully examined coefficients and p-values in models with VIF > 3 (Zuur et al. 2010). For models with interactions, we excluded the interaction term and tested the additive models' VIF. Through this diagnostic process we found relatively high VIF scores for covariates in the top-ranked models for Shinumo Creek (e.g., rainbow trout abundance VIF = 13.4) and Havasu Creek growth (e.g., season VIF = 6.1). We found that by removing the random intercept for translocation year, VIF scores decreased to < 2.5, and the p-values and SEs for coefficients in the top-models with and without translocation year as a random effect showed little difference, suggesting a minimal effect of collinearity. The top-ranked growth models for the combined dataset for both streams had VIF scores < 3 (combined dataset VIF < 2.76). Model fit was further by examining model residuals (Fig. S4 – S6).

Survival and Emigration – We used a joint live-recapture/live-resight (JLRR) model to estimate true survival (S_i, probability of survival through interval i) and site fidelity (F_i) of translocated humpback chub (Barker 1997). This model is particularly useful for determining the fate of translocated individuals because it can incorporate continuously-collected data from PIAs and captures throughout the CRE during GCDAMP-interagency monitoring, which we considered "resights," as well as recaptures during targeted monitoring within translocations

sites (e. g., Horton and Letcher 2008, Conner et al. 2015). Additional parameters estimated by the JLRR model include recapture probability (p_j) during translocation site monitoring events, resight probability outside of translocations sites (R_i, i.e., probability of detection during the interval *i*), temporary emigration (F'_j, the probability a fish is not available for capture during *j* sampling event, but is available at *j*+1), the probability of resighting before an individual dies during the interval (R'_i), and the probability an animal is found dead during the interval (r_i).

For Shinumo Creek humpback chub *S* and *F* models, we included re-captures during summer and fall netting events between June, 2009 and June, 2014, and resights from the Shinumo PIA and CRE between recapture events from June, 2009 to May, 2019. Encounter histories were developed to represent positive and negative observations for individual fish, and those recaptured or resighted multiple times during a monitoring event or interval between events were only recorded once (i.e., “present”). We coded Shinumo PIA detections that occurred during the recapture sampling events (i.e., the ~5-7 days during summer and fall sampling) as recaptures. Following the extirpation of humpback chub from Shinumo Creek in July 2014, zero recaptures occurred, but we created “dummy” post-flood recapture events with fixed p =1, assuming certainty of humpback chub extirpation. Creating dummy recapture events enabled the use of all available resight data from the CRE through 2019 to estimate monthly *S* of humpback chub that had emigrated from Shinumo Creek prior to the flood event, and assess the impact of the flood on *S*.

We estimated *S* and *F* for Havasu Creek humpback chub in a similar fashion, except no PIA detections at the mouth of Havasu Creek were available to augment resight encounter histories. Resights from GCDAMP monitoring trips between June, 2011 and August, 2019 from anywhere in the CRE, and recaptures from within Havasu Creek during spring and fall NPS

monitoring trips between June, 2011, and October, 2019, were incorporated into the JLRR model. We also defined two groups (*g*) of humpback chub in Havasu Creek, including translocated and non-translocated fish (either fish produced *in situ*, or immigrated during elevated 2011 Colorado River discharge; Healy et al. 2020a), for estimation of *S* and *F*. We attempted to fit and age-cohort model, as described in Cooch and White (2011), to estimate survival of newly translocated humpback chub during the first two intervals following translocation; however, the data did not allow for estimation of most *S* parameters using the age-cohort structure.

Our JLRR model selection process proceeded in stages, where we began by finding the best supported structure on recapture and resight probabilities (*p, R, R'*), using combinations of time-varying and constant parameters, while maintaining flexibility on *S* and *F* (i.e., S_t, or $S_{g \times t}$, F_t). We assumed *R* and *p* probabilities were equal for both translocated and non-translocated groups in Havasu Creek based on previous analyses (Healy et al. 2020). We set $r_i = 0$, since only 5 individuals (<0.002% of translocated fish) were found dead during our study. We tested for permanent emigration of humpback chub from Shinumo Creek if an individual was detected on the PIA (cf. Spurgeon et al. 2015), which we accomplished by comparing a constant and time-varying *F'* to models with *F'*= 0. We assumed permanent emigration (*F'*= 0) in Havasu Creek models due to the presence of barriers near the mouth (see Healy et al. 2020). Using the established parsimonious model of *p*, *R,* and *R'* probabilities from the first model-selection stage, we compared combinations of models with time-varying, constant, and group-specific *F* and then *S*, while holding the other parameter flexible (F_t or S_t). Finally, we combined the most-supported model structure for *p, R, R'*, *S,* and *F*, and then added combinations of environmental and biological covariates to *S* and *F* parameters. Ultimately, we retained flexibility (*t*) in *p, R, S,* and

F, and *R'* was held constant prior to the addition of covariates to *F* and *S*. We constructed and ranked models using Program MARK (White and Burnham 1999) and Akaike Information Criteria adjusted for small sample sizes (AIC_c, Burnham & Anderson 2002).

We opted to reduce dimensionality in our covariate data by using principle component analysis (PCA) to represent environmental and biological variation (Graham 2003), given correlations among covariates. This also avoided complications typically arising from the almost infinite number of hydrologic metrics available for assessment, which are often correlated (Yarnell et al. 2020). We included combinations of the first two principal components (PCs), which are orthogonal vectors derived from linear combinations of covariates, representing 42% and 23% of variation in the covariates in Havasu Creek, and 51.3% and 22.0% in Shinumo Creek, as explanatory variables in JLRR models (Figure 2). For Havasu Creek, PC1 represented variation in flood magnitude and frequency and temperature (−), and PC2 represented indices of abundance for humpback chub and the number of translocated chub (−), rainbow trout abundance (+), and the timing of large (> 28 m^3/s) floods relative to translocation timing (−, Figure 2). PC1 for Shinumo Creek represented a gradient of rainbow trout, speckled dace, and humpback chub abundance (−), and the total acres of fire below the canyon rim in the watershed (+). Shinumo Creek PC2 represented flood-pulse frequency (−). The spatial and temporal distribution of sampling monitoring effort generating data for the JLRR model is depicted in Fig. S3.

Recruitment – To assess drivers of annual recruitment rates for humpback chub in Havasu Creek, we used a temporal symmetry model (TSM; Pradel 1996). The TSM is an open-population model that simultaneously estimates apparent survival (φ, confounded by emigration) using

individual encounter histories, and estimates the relative contributions of adult survival and recruitment (ρ) towards the population growth rate (λ) that is interpretable through a "seniority probability" (γ) parameter estimated using the time-reversed encounter history (Pradel 1996, Nichols et al. 2000, Budy et al. 2017). Assumptions of the TSM, in addition to assumptions of typical demographically-open models (Lebreton et al. 1992), include the size of the study area and sampling effort are held constant to avoid biasing ρ. We restricted our TSM analysis to mark-recapture data collected during spring trips when 2 sampling passes were consistently conducted. Effort differed slightly during the May 2014 sampling event, relative to other events, when logistical delays hindered our ability to complete both passes throughout the entire study site (Healy et al. 2020). Nonetheless, 2-passes were completed in the stream reach where >85% of humpback chub captures have been recaptured (2,915/3,390 total captures), and thus, we assumed this discrepancy would result in little bias in our ρ estimates.

We were interested in TSM estimates for only non-translocated fish, which we separated from translocated cohorts by defining representative groups in the encounter history matrix. Assigning individuals to groups (translocated and non-translocated) allowed us to share p_j from both groups if appropriate (i.e., if no group-level differences in p_j were found), while generating group-specific estimates of ρ and γ. In the TSM, recruitment is defined as the number of new adults at time $t + 1$ relative to the number of adults at time t, and we considered newly PIT-tagged fish as recruits. The average size at tagging was 204 mm TL, which corresponded to approximately age-2 (Healy et al. 2020) when fish begin to become mature (i.e., defined as fish in spawning condition; size at tagging, Figure S2). We ignored estimates of λ, ρ, and γ for the translocated group because those parameters were directly related to translocations. We used the $\varphi\rho p$ and the $\varphi\gamma p$ parameterizations of the TSM in Program MARK (White and Burnham 1999)

to construct multiple models with combinations of constant and time-varying parameters, including comparisons of group, time-varying, and constant *p*, to generate recruitment estimates and assess the relative contributions of adult survival and recruitment to population growth. We considered estimates of $\gamma > 0.5$ to indicate greater influence of ρ on λ, while $\gamma < 0.5$ indicated φ was more important for λ in a given year (Budy et al. 2017). To test for, and adjust for overdispersion in our TSM, we used a goodness-of-fit (median $\hat{c}$) bootstrapping procedure in Program MARK using a fully-parameterized CJS model ($\varphi_{g \times t}\, p_t$). If estimated median $\hat{c}$ was >1 but < 3 (Lebreton et al. 1992), we made adjustments to account for $\hat{c}$ before final model selection (i.e., quasi-AIC$_c$ [QAIC$_c$] values were computed with the estimated median $\hat{c}$ value).

Given constraints related to annual time intervals and our inability to differentiate between seasonal variation, we limited are hypothesis testing to annual drivers of recruitment during early life stages. We tested covariates including flooding-pulse frequency, and humpback chub and rainbow trout abundance indices during the natal year, as drivers of recruitment. The humpback chub abundance index metric differed slightly from the metric used for survival hypothesis testing, in that we summed the number of humpback chub translocated and captured in the spring of the natal year for each cohort, which we defined as recruitment year $t - 2$. The number of rainbow trout captured in spring, and flood-pulse frequency during the summer of the natal year were also tested.

Table S1. Summary of average size, tag dates, release dates, and number of Humpback chub released into Shinumo and Havasu creeks from 2009-2018 (see Spurgeon et al. 2015, Schelly et al. 2019, Healy et al. 2020).

Tributary	Hatchery Tagging Date	Average Length (mm)	Average Weight (g)	Release Date	Number Translocated
Shinumo Creek	May 18, 2009	127.9	18.7	June 15, 2009	302
Shinumo Creek	June 10, 2010	121.1	15.3	June 23, 2010	300
Shinumo Creek	May 5, 2011	88.9	5.4	June 21, 2011	300
Shinumo Creek	June 10, 2013	123.3	14.8	June 15, 2013	200
Havasu Creek	May 5, 2011	86.1	4.8	June 28, 2011	243
Havasu Creek	May 10, 2012	124.7	16.7	May 13, 2012	298
Havasu Creek	May 9, 2013	123.1	14.9	May 14, 2013	300
Havasu Creek	May 14, June 5, 2014	123.5	16.4	May 14, 2014, June 5, 2014	300, 209
Havasu Creek	May 13, 2015	131	20.3	May 20, 2015	300
Havasu Creek	May 10, 2016	130	18.5	May 18, 2016	305
Bright Angel Creek	May 1, 2014	258	141	May 14, 2018	116*

*A hatchery tagging error led to an uncertain number of uniquely tagged fish released.

Table S2. Somatic growth models and AICc ranking for humpback chub translocated to Shinumo and Havasu Creek (modeled using lme4). The top model is indicated by a ΔAIC_c =0, and 2 models that did not converge for Shinumo Creek are indicated by “NC”. Key (z-scored) covariates for hypothesis testing included flood-pulse frequency (z_Flood), rainbow trout (z_RBT) and humpback chub abundance (z_HBC).

Model	variables	Shinumo Creek ΔAIC_c	Havasu Creek ΔAIC_c
m0	growth_rate ~ Length.mm.+season+(1| PITTAG)+(1| transl.year)	28.4	383.4
m1	growth_rate ~ Length.mm.+season+z_Flood+(1| PITTAG)+(1| transl.year)	16.7	30.2
m2	growth_rate ~ Length.mm.+season+z_RBT+(1| PITTAG)+(1| transl.year)	29.6	115.2
m3	growth_rate ~ Length.mm.+season+z_Flood*z_HBC+(1| PITTAG)+(1| transl.year)	3.3	4.8
m4	growth_rate ~ Length.mm.+season+z_Flood*z_RBT+(1| PITTAG)+(1| transl.year)	NC	25.5
m5	growth_rate ~ Length.mm.+season*z_Flood+(1| PITTAG)+(1| transl.year)	8.7	5.5
m6	growth_rate ~ Length.mm.+season*z_Flood+z_HBC+(1| PITTAG)+(1| transl.year)	2.4	0
m7	growth_rate ~ Length.mm.+season*z_Flood+z_RBT+(1| PITTAG)+(1| transl.year)	10	6.5
m8	growth_rate ~ Length.mm.+season+z_Flood+z_HBC+(1| PITTAG)+(1| transl.year)	2.5	24.1
m9	growth_rate ~ Length.mm.+season+z_Flood+z_RBT+(1| PITTAG)+(1| transl.year)	9.1	32.1
m10	growth_rate ~ Length.mm.+season*z_HBC+z_Flood+(1| PITTAG)+(1| transl.year)	1.6	18.4
m11	growth_rate ~ Length.mm.+season*z_RBT+z_Flood+(1| PITTAG)+(1| transl.year)	0	23.8
m12	growth_rate ~ Length.mm.+season*z_HBC +(1| PITTAG)+(1| transl.year)	1.2	34.5
m13	growth_rate ~ Length.mm.+season*z_RBT+(1| PITTAG)+(1| transl.year)	8.7	108.9
m14	growth_rate ~ Length.mm.+ season + z_HBC +(1| PITTAG)+(1| transl.year)	NC	35.9

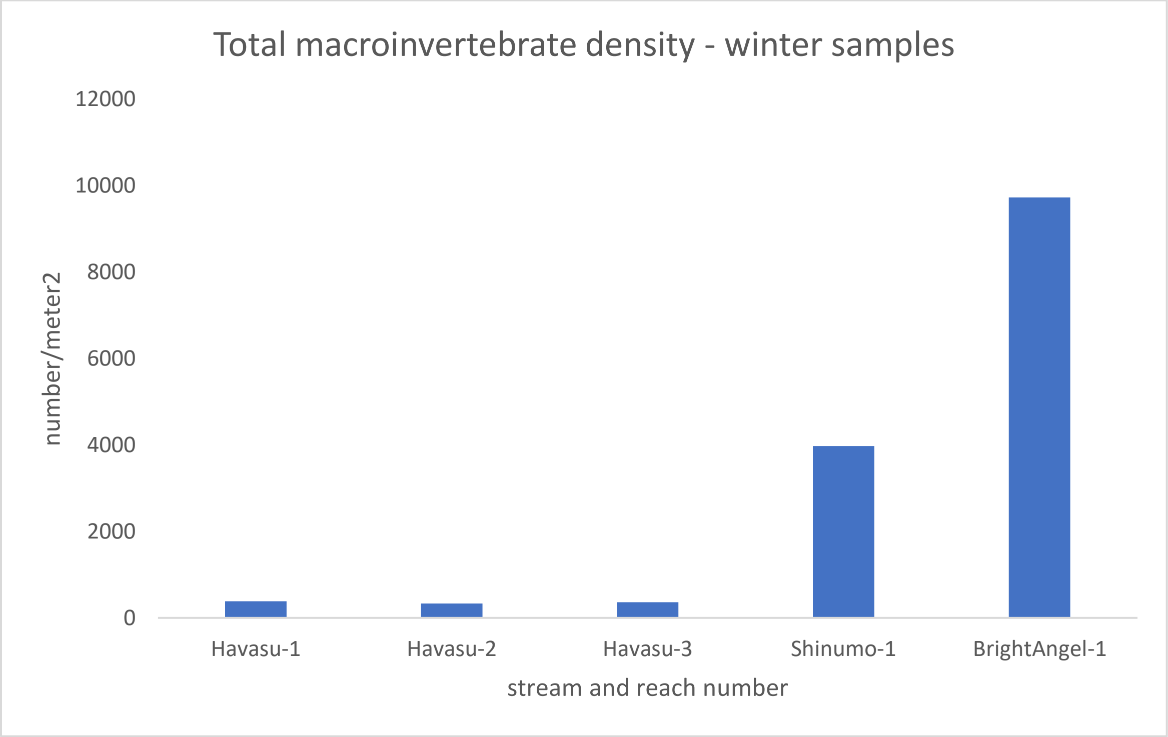

Figure S1. Macroinvertebrate density in translocation sites, from samples collected between 2011 and 2019.

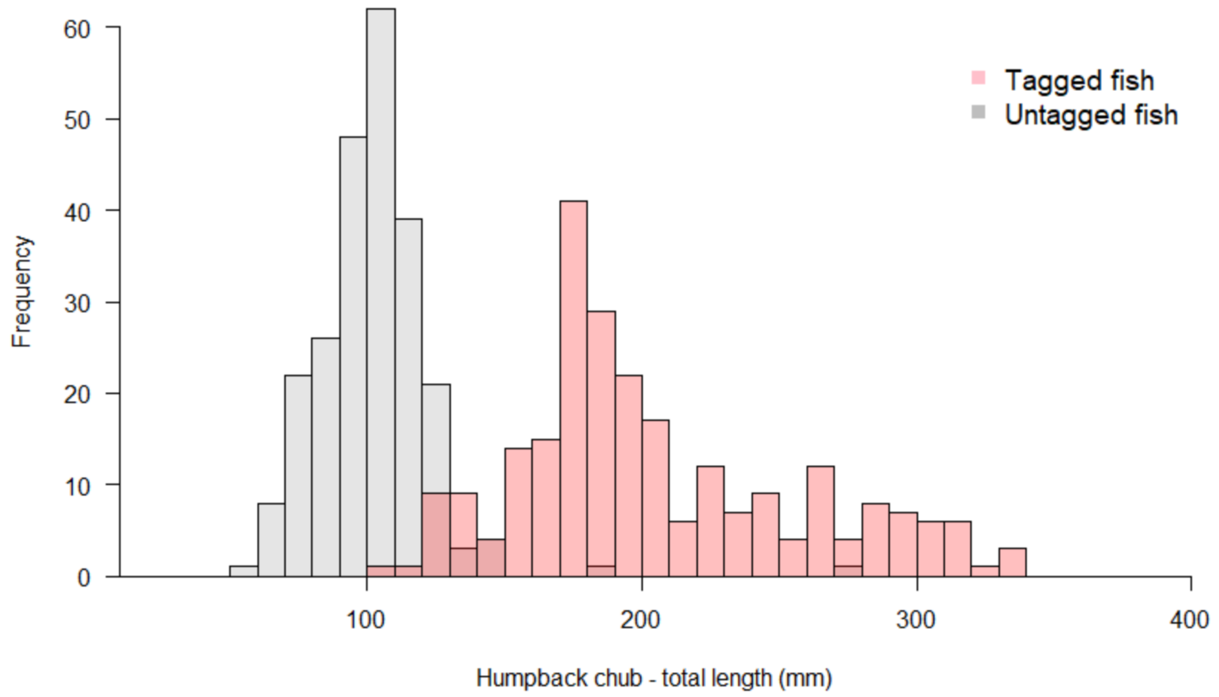

Figure S2. Havasu Creek tagging size - referenced in the Pradel model section- non-translocated fish.

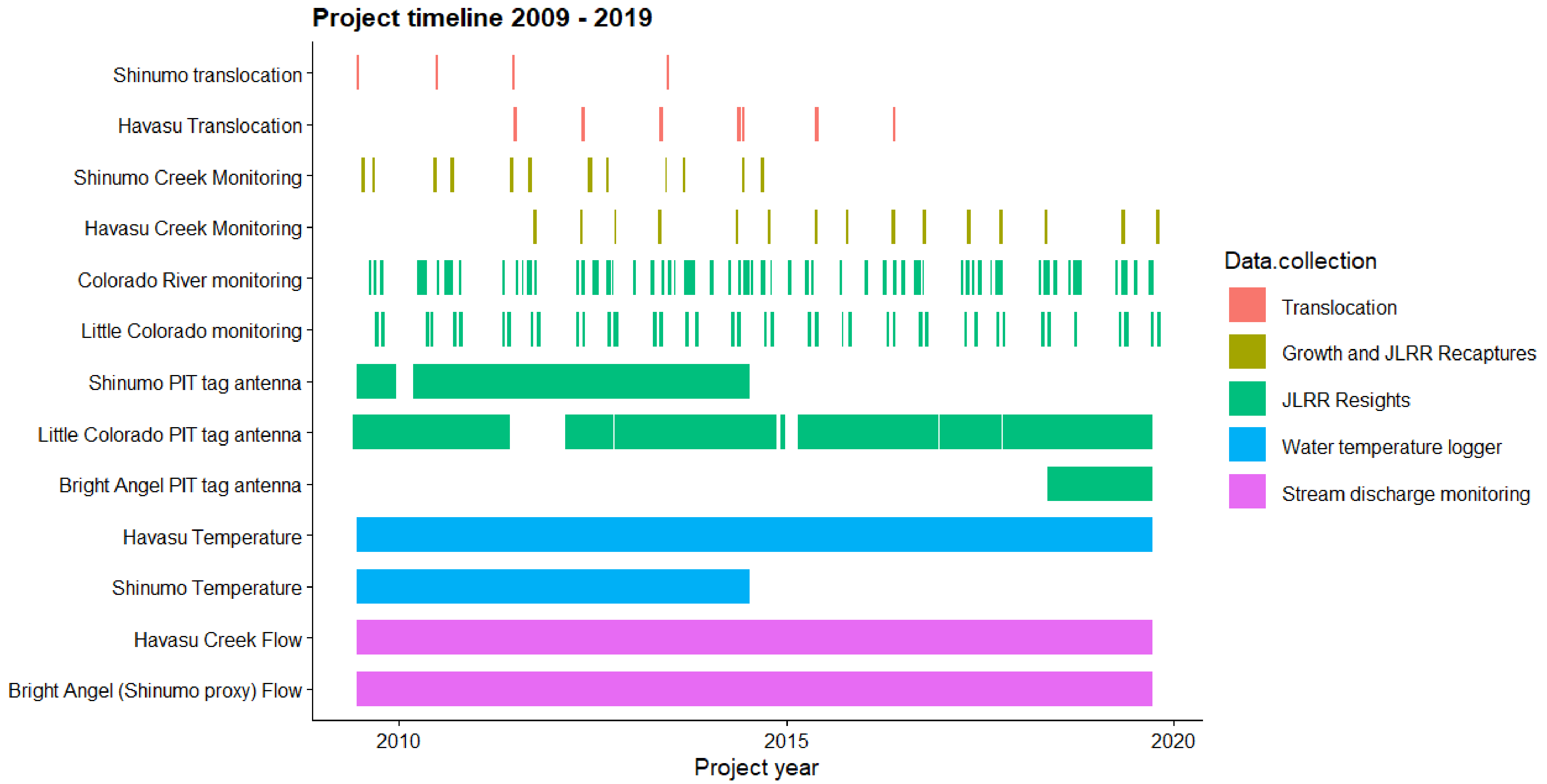

Figure S3. Timing of translocations to Shinumo and Havasu creeks, and the distribution of sampling by location and type. Sampling occasions color-coded as "Growth and JLRR Recaptures" included sampling within translocation sites to generate data for somatic growth modeling and physical recapture data to populate encounter histories for the JLRR model and TSM model (Havasu spring trips only). Samples coded as "JLRR resights" included capture (any gear type) or antenna detection data from outside translocation sites in the Colorado River ecosystem (including other tributaries). Gaps in PIT tag antenna coverage over time depict periods when flooding or power loss occurred; however, timing for the Little Colorado River antenna is approximate (data for actual operational periods between 2009-2011 were unavailable; see Pearson et al. 2015). Water temperature loggers were lost and the PIT tag antenna was destroyed during summer 2014. Abundance indices representing humpback chub, rainbow trout, or speckled dace were derived from catch data collected during JLRR recapture occasions, and applied to the previous interval for survival and somatic growth models.

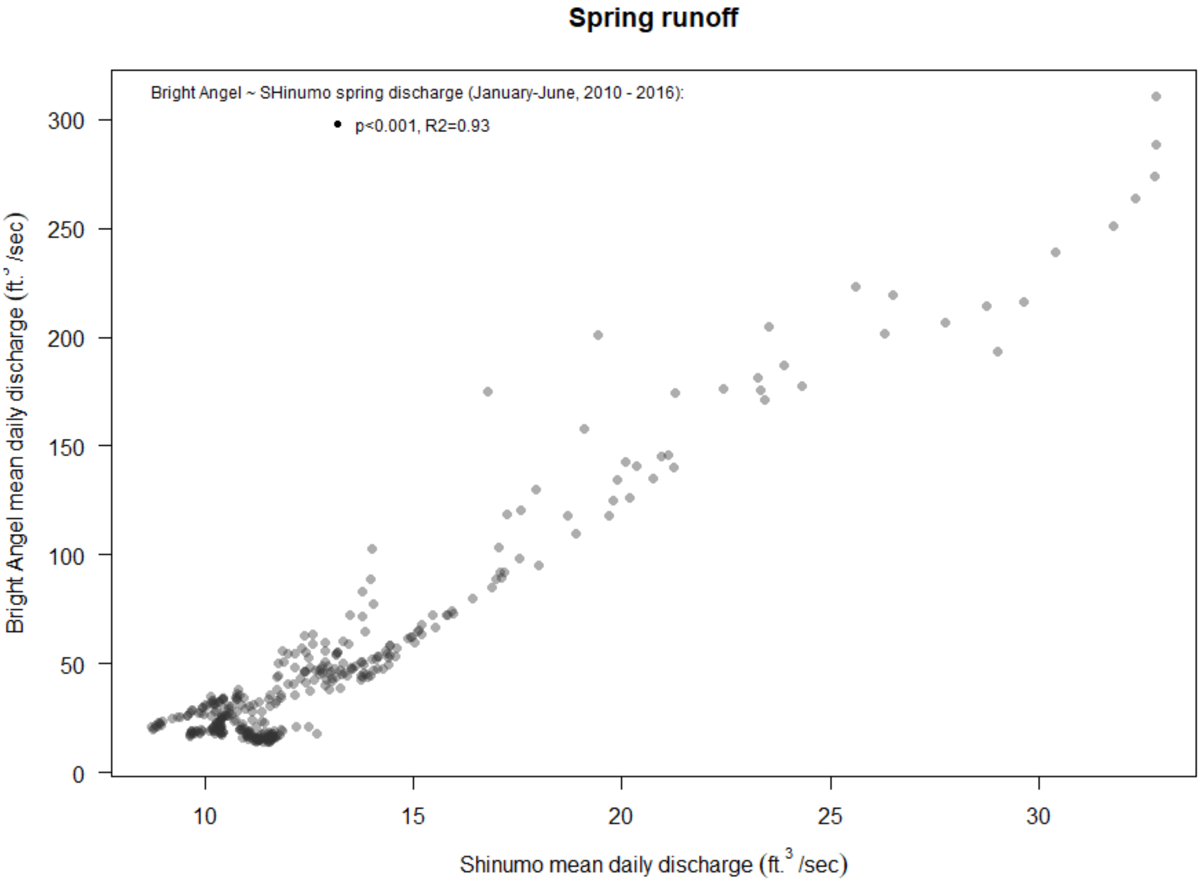

Figure S4. Correlation between available Shinumo Creek discharge data and Bright Angel Creek discharge data (USGS gaging station 9403000) during spring (January – June, 2010-2016). We used Bright Angel Creek hydrology data to calculate representative flood-pulse frequency for Shinumo Creek. Both streams flow from the North Rim of Grand Canyon National Park, and have similar watershed characteristics (forest type cover, elevation, climate, groundwater-fed). Few data were available for summer monsoon season from Shinumo Creek for comparison to Bright Angel Creek during the same season, due to damage to equipment related to floods. Remoteness of the site prevented crews from maintaining the stream gage outside of June and September.

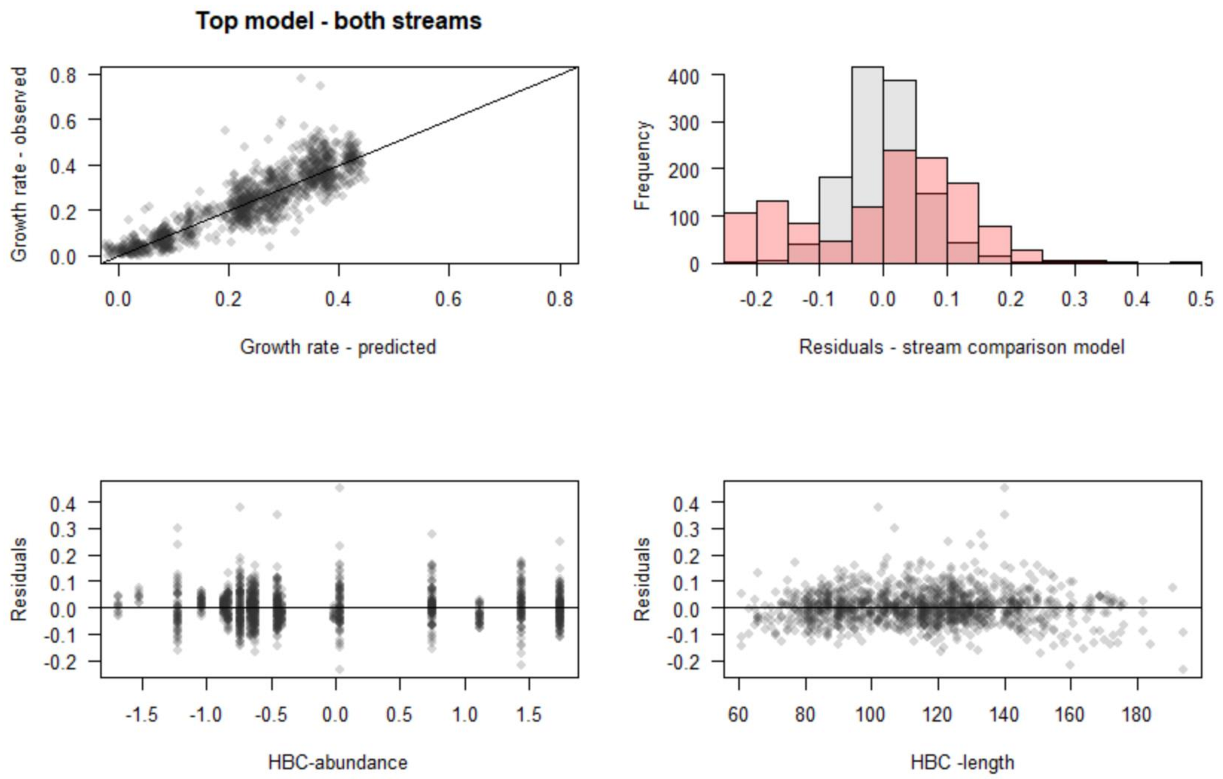

Figure S5. Model diagnostics/fit statistics for the top model (# g6y1) for growth including data for all streams/cohorts.

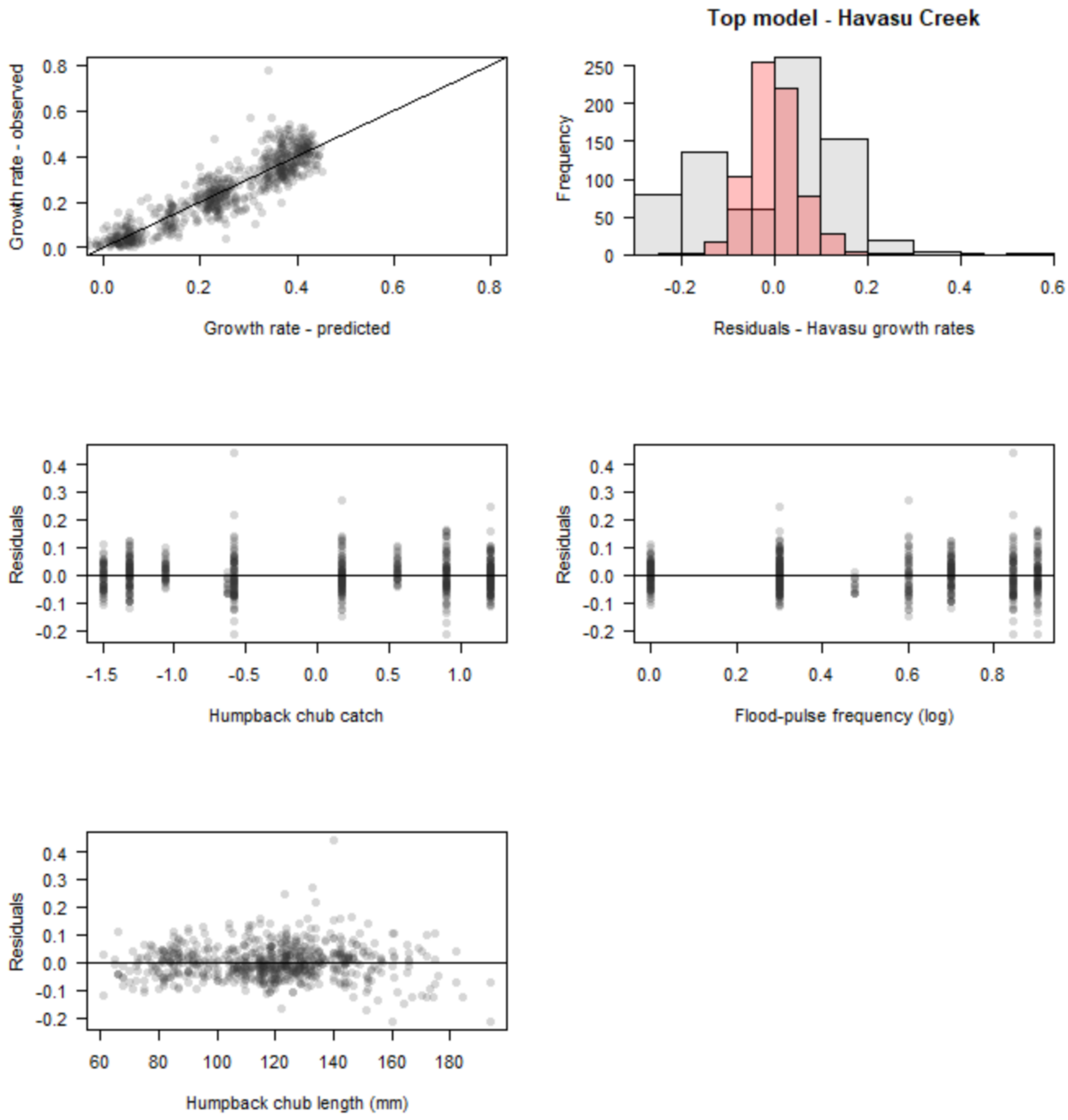

Figure S6. Model diagnostics for top model for Havasu Creek growth.

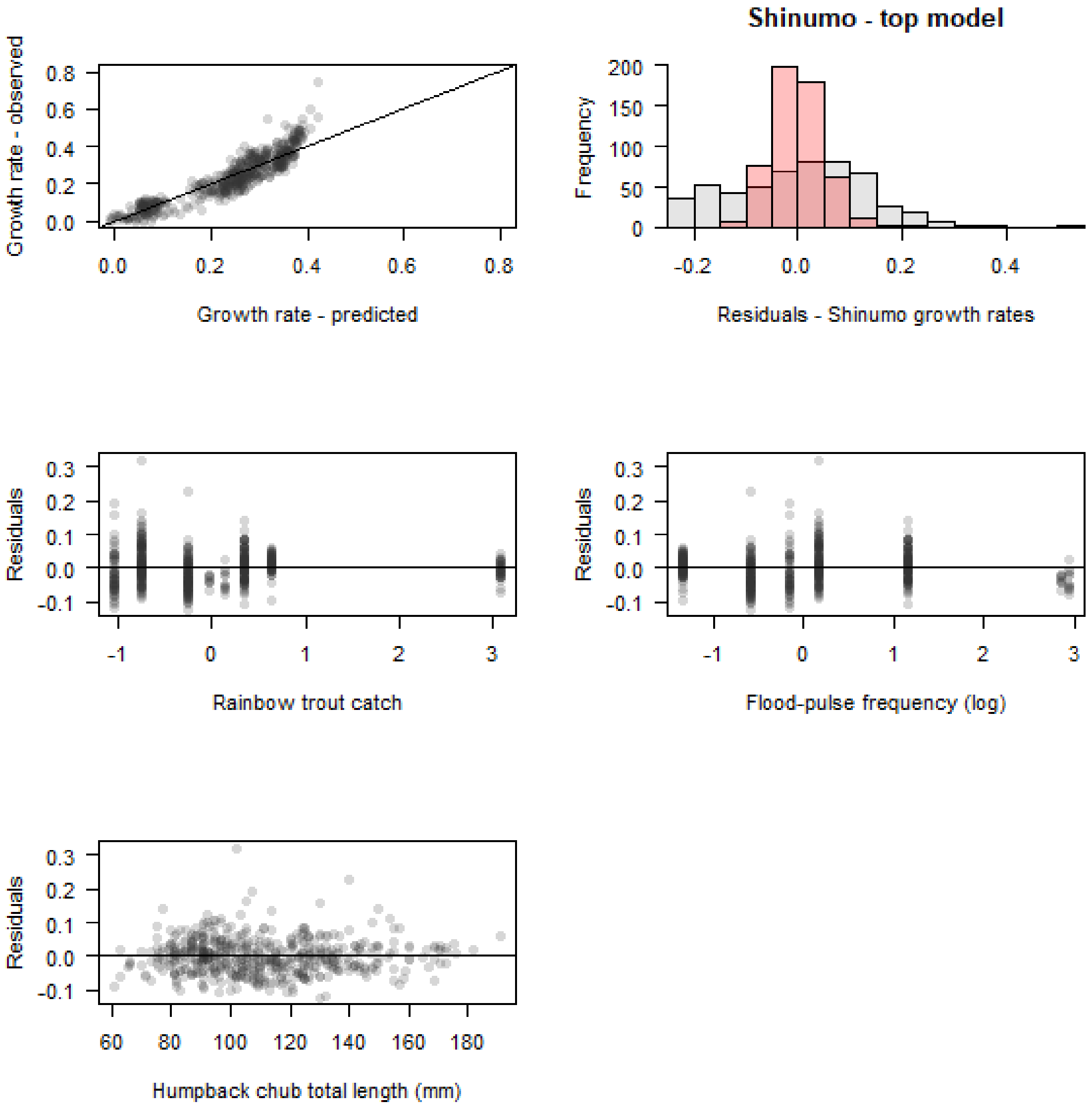

Figure S7. Model diagnostics for Shinumo Creek growth rates.

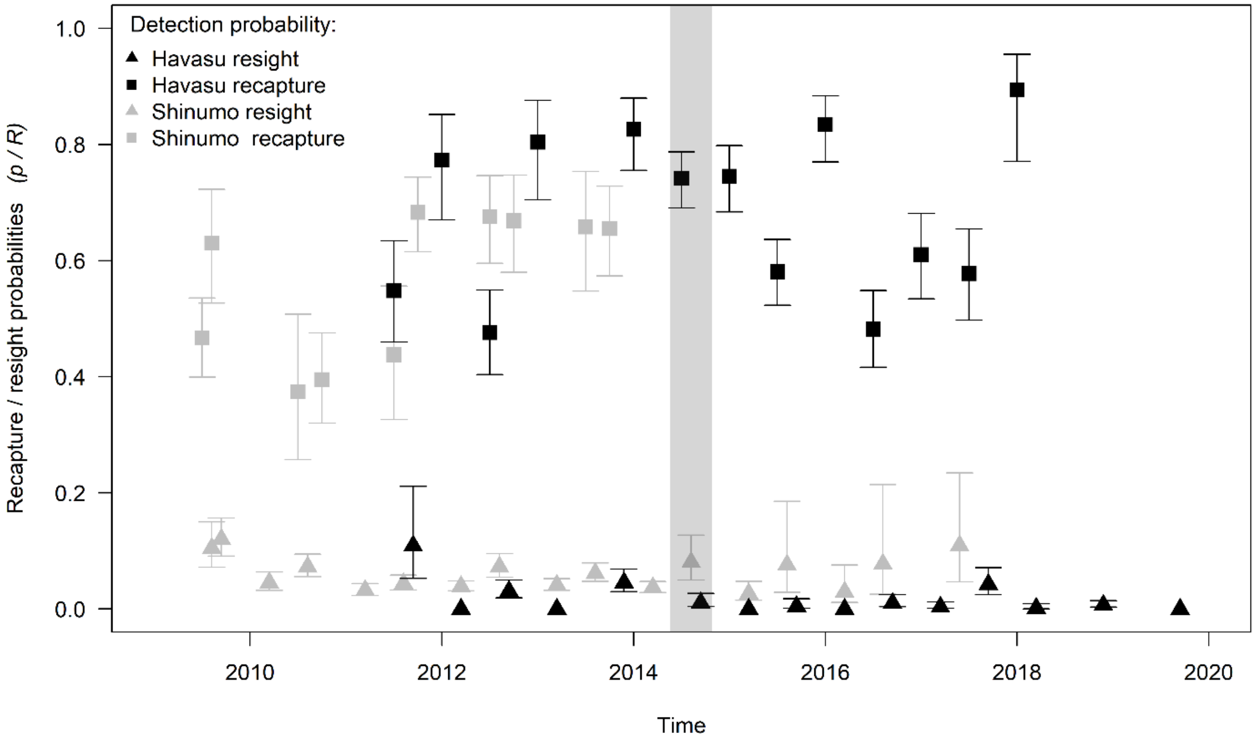

Figure S8. Recapture/resight probabilities from Havasu Creek joint live-recapture/resight model. Note the last estimate of R and p are confounded.

Table S3. Supplementary info PCA scores for Havasu Creek environmental covariates.

Variable	Label	PC1	PC2	PC3
Number of translocated humpback chub	No.Transl	-0.23	**-0.46**	**0.41**
Number of days after translocation until flood > 1000 cfs/28 cms	No.daysto1000	-0.03	**-0.44**	**0.45**
Humpback chub catch (index of abundance)	HBC.catch	0.13	**-0.59**	-0.14
Flood-pulse frequency (log-transformed)	Floodpulse	**-0.47**	-0.03	-0.07
Rainbow trout catch (index of abundance)	RBT.catch	0.08	**0.44**	**0.49**
Speckled dace catch (index of abundance)	SPD.catch	0.13	-0.18	**-0.58**
Cumulative degree-days	cDD	**-0.49**	-0.03	-0.02
Maximum flood	maxFlood	**-0.46**	0.05	-0.15
Number of days discharge > 1000 cfs/28 cms	days.ov.1000	**-0.48**	0.11	-0.10
Principle component statistics				
Proportion of Variance		0.42	0.23	0.18
Cumulative Proportion		0.42	0.65	0.83

Literature Cited

Barker, R. J. 1997. Joint modeling of live-recapture , tag-resight , and tag-recovery data. Biometrics 53:666–677.

Bates, D., M. Maechler, B. Bolker, and S. Walker. 2015. Fitting linear mixed effects models using lme4. Journal of Statistical Software 67:1–48.

Bolker, B., and R. D. C. Team. 2017. bblme: tools for general maximum likelihood estimation.

Budy, P. E., T. Bowerman, R. Al-Chokhachy, M. Conner, and H. Schaller. 2017. Quantifying long-term population growth rates of threatened bull trout: challenges, lessons learned, and opportunities. Canadian Journal of Fisheries and Aquatic Sciences 74:2131–2143.

Burnham, K. P., and D. R. Anderson. 2002. Model selection and multimodel inference: a practical information-theoretic approach. 2nd editio. Springer-Verlag New York, Inc., New York.

Conner, M. M., S. N. Bennett, W. C. Saunders, and N. Bouwes. 2015. Comparison of tributary survival estimates of steelhead using Cormack–Jolly–Seber and Barker models: implications for sampling efforts and designs. Transactions of the American Fisheries

Society 144:34–47.

Cooch, E., and G. White. 2011. Program MARK: a gentle introduction. 9th Editio. http://www.phidot.org/software/mark/docs/book/.

Dormann, C. F., J. Elith, S. Bacher, C. Buchmann, G. Carl, G. Carré, J. R. G. Marquéz, B. Gruber, B. Lafourcade, P. J. Leitão, T. Münkemüller, C. Mcclean, P. E. Osborne, B. Reineking, B. Schröder, A. K. Skidmore, D. Zurell, and S. Lautenbach. 2013. Collinearity: A review of methods to deal with it and a simulation study evaluating their performance. Ecography 36:27–46.

Dzul, M. C., C. B. Yackulic, J. Korman, M. D. Yard, and J. D. Muehlbauer. 2017. Incorporating temporal heterogeneity in environmental conditions into a somatic growth model. Canadian Journal of Fisheries and Aquatic Sciences 74:316–326.

Fox, J., and S. Weisberg. 2014. An R companion to applied regression. Page Robust Regression in R. Third edit. Sage, Thousand Oaks, California.

Gelman, A., and J. Hill. 2009. Data analysis using regression and multilevel/ hierarchical models. Cambridge University Press, New York, New York.

Graham, M. H. 2003. Confronting multicollinearity in ecological multiple regression. Ecology 84:2809–2815.

Healy, B. D., E. C. Omana Smith, R. C. Schelly, M. A. Trammell, and C. B. Nelson. 2020. Establishment of a reproducing population of endangered humpback chub through translocations to a Colorado River tributary in Grand Canyon, Arizona. North American Journal of Fisheries Management 40:278–292.

Horton, G. E., and B. H. Letcher. 2008. Movement patterns and study area boundaries: Influences on survival estimation in capture-mark-recapture studies. Oikos 117:1131–1142.

Lebreton, J. D., K. P. Burnham, J. Clobert, and D. R. Anderson. 1992. Modeling survival and testing biological hypotheses using marked animals: a unified approach with case studies. Ecological Monographs 62:67–118.

Lüdecke, D. 2019. sjPlot: Data visualization for statistics in social science.

Nichols, J. D., J. E. Hines, J. Lebreton, and R. Pradel. 2000. Estimation of contributions to population growth : a reverse-time capture-recapture approach. Ecology 81:3362–3376.

Pearson, K. N., W. L. Kendall, D. L. Winkelman, and W. R. Persons. Evidence for Skipped Spawning in a Potamodromous Cyprinid, Humpback Chub (*Gila Cypha*), with Implications for Demographic Parameter Estimates. Fisheries Research 170 (2015): 50–59.

Pine, W. E., B. S. Gerig, and C. Finch. 2017a. Characterizing growth and condition of endangered humpback chub in the Lower Colorado River.

Pine, W. E., K. Limburg, B. S. Gerig, C. Finch, D. Chagaris, L. Coggins, D. Speas, and D. A. Hendrickson. 2017b. Growth of endangered humpback chub in relation to temperature and

discharge in the Lower Colorado River. Journal of Fish and Wildlife Management 8:322–332.

Pradel, R. 1996. Utilization of capture-mark-recapture for the study of recruitment and population growth rate. Biometrics 52:703–709.

R Core Team. 2019. R: A language and environment for statistical computing. R foundation for statistical computing, Vienna, Austria.

Schelly, R., B. Healy, E. O. Smith, R. Koller, A. Bachelier, and A. Martin. 2019. Bright Angel Creek humpback chub translocation, May 13-16, 2018 trip report. National Park Service Report Prepared for the Upper Colorado Region, Bureau of Reclamation, Interagency Agreement Number: R14PG00051.

Spurgeon, J. J., C. P. Paukert, B. D. Healy, M. Trammell, D. Speas, and E. Omana Smith. 2015. Translocation of humpback chub into tributary streams of the Colorado River: implications for conservation of large-river fishes. Transactions of the American Fisheries Society 144:502–514.

Weisberg, S., G. Spangler, and L. S. Richmond. 2010. Mixed effects models for fish growth. Canadian Journal of Fisheries and Aquatic Sciences 67:269–277.

White, G. C., and K. P. Burnham. 1999. Program mark: Survival estimation from populations of marked animals. Bird Study Supplement:S120-139.

Yarnell, S. M., E. D. Stein, J. A. Webb, T. Grantham, R. A. Lusardi, J. Zimmerman, R. A. Peek, B. A. Lane, J. Howard, and S. Sandoval-Solis. 2020. A functional flows approach to selecting ecologically relevant flow metrics for environmental flow applications. River Research and Applications 36:318–324.

Zuur, A. F., E. N. Ieno, and C. S. Elphick. 2010. A protocol for data exploration to avoid common statistical problems. Methods in Ecology and Evolution 1:3–14.
